# Small leucine rich proteoglycans, a novel link to osteoclastogenesis

**DOI:** 10.1038/s41598-017-12651-6

**Published:** 2017-10-03

**Authors:** Vardit Kram, Tina M. Kilts, Nisan Bhattacharyya, Li Li, Marian F. Young

**Affiliations:** 1 Craniofacial and Skeletal Diseases Branch, National Institute of Dental and Craniofacial Research, National Institutes of Health, Bethesda, MD 20892 USA; 2 Scientific Review Branch, Division of Extramural Activities, National Institute of Dental and Craniofacial Research, National Institutes of Health, Bethesda, MD 20892 USA

## Abstract

Biglycan (Bgn) and Fibromodulin (Fmod) are subtypes of the small leucine-rich family of proteoglycans (SLRP). In this study we examined the skeletal phenotype of BgnFmod double knockout (*BgnFmod* KO) mice and found they were smaller in size and have markedly reduced bone mass compared to WT. The low bone mass (LBM) phenotype is the result of both the osteoblasts and osteoclasts from *BgnFmod* KO mice having higher differentiation potential and being more active compared to WT mice. Using multiple approaches, we showed that both Bgn and Fmod directly bind TNFα as well as RANKL in a dose dependent manner and that despite expressing higher levels of both TNFα and RANKL, *BgnFmod* KO derived osteoblasts cannot retain these cytokines in the vicinity of the cells, which leads to elevated TNFα and RANKL signaling and enhanced osteoclastogenesis. Furthermore, adding either Bgn or Fmod to osteoclast precursor cultures significantly attenuated the cells ability to form TRAP positive, multinucleated giant cells. In summary, our data indicates that Bgn and Fmod expressed by the bone forming cells, are novel coupling ECM components that control bone mass through sequestration of TNFα and/or RANKL, thereby adjusting their bioavailability in order to regulate osteoclastogenesis.

## Introduction

As life expectancy continues to rise, the burden of age-related diseases is expected to increase. One such age-related disease is osteoporosis. The skeleton is a dynamic tissue undergoing continuous remodeling - old bone is being resorbed by osteoclasts and new bone is laid down by osteoblasts- at multiple foci at the same time. When this well synchronized process becomes unbalanced, the strength and integrity of bone is compromised. Osteoporosis is defined as a condition in which the bone mass is reduced, either by a lower rate of new bone formation, by an enhanced process of bone resorption, or a combination of both, ultimately leading to weak and fragile bones that tend to break. Understanding more about how the bone turnover process is synchronized will help in the generation of novel therapies to ameliorate age related osteopenia.

Osteoclasts originate from myeloid/monocytic hematopoietic precursors^1^. The fusion and differentiation of mononuclear precursor cells into multinucleated bone-resorbing osteoclasts relies mainly on the presence and availability of macrophage colony-stimulating factor (M-CSF) and receptor activator of NF-κB ligand (RANKL), both of which are secreted by osteogenic precursors and osteoblasts/osteocytes^2–5^. A major osteoclastogenesis differentiation factor, RANKL, works through the type I transmembrane receptor of the TNFα family, RANK. At early stages of osteoclast differentiation, M-CSF promotes the expression of RANK by myeloid progenitors, further increasing the number of RANK–expressing cells and priming them to react to RANKL^6^.

Tumor necrosis factor alpha (TNFα) is a multifunctional proinflammatory cytokine produced by a wide variety of immune and epithelial cell types. It plays a central role in inflammation, apoptosis, and immune system development. TNFα is synthesized intracellularly as a 26-kDa membrane-bound homotrimer (pro-TNFα) that is then cleaved to release a soluble 17-kDa trimeric molecule. The soluble molecule is then able to bind either the ubiquitous TNFR1 or the hematopoietic cell-restricted TNFR2. Both membrane and soluble forms of TNFα are biologically active. RANKL as well as osteoprotegerin (OPG) are type 2 protein members of the TNFα superfamily, whereas RANK is a type 1 transmembrane protein of the TNF receptor family^7^. TNFα has been shown to lead to differentiation and survival of osteoclasts in a RANKL independent manner^8,9^. Like M-CSF, in addition to promoting the expression of RANKL, TNFα also promotes the expression of RANK by myeloid progenitors further increasing their ability to respond to RANKL^10^. Additionally, TNFα has been shown to induce both M-CSF and RANKL expression in bone marrow stromal cells (BMSCs), directly activating osteoclast precursors and contributing to their differentiation^10–12^.

Small leucine rich proteoglycans (SLRPs) are widely expressed macromolecules found abundantly in the extraceullular matrix (ECM). SLRPs consist of a relatively small protein core of about 40–60 kDa, most of which contains 10–12 motifs of leucine rich repeats (LRR), 20–30 amino acid long^13,14^. Based on their amino-acid sequence, the difference in the spacing of the N-terminal cysteine residues, and the intron-exon organization, they were divided into five subclasses^15^. All the canonical SLRPs undergo post translational modification in the form of glycosaminoglycan (GAG) chain attachments. Biglycan (Bgn), a class I SLRP, has two chondroitin and/or dermatan sulfate side chains^16^, whereas fibromodulin (Fmod), a class II SLRP, has 5 potential sites for keratan sulfate chains attachment^17,18^. The first function recognized for SLRPs was their ability to regulate collagen fibril size and assembly. The core proteins of SLRPs directly bind to collagen molecules through the LRR motifs, limiting the diameter of the mature collagen fiber^19,20^. In addition, the various combinations of protein cores substituted with one or more GAG chains of varying types and glycosylation states, along with their pericellular localization, enables SLRPs to interact with a multitude of cell surface receptors, cytokines, chemokines, growth factors and other ECM components in a multivalent capacity. The direct binding of these molecules to sites on the core protein or to the GAG chains controls their bioavailability and activity, either by (1) sequestering them in the ECM thus creating a biological gradient or reservoir close to the cell surface, (2) presenting them to their individual receptors, therefore enhancing the signaling aptitude or (3) keeping them away from the relevant receptors, by preventing the ligand-receptor interaction thus attenuating signal transduction^13,21,22^. All these interactions have a direct effect on cell-signaling pathways regulating proliferation, migration and the complement immune system^23–26^.

Animals deficient in type I and II SLRPs show, among other phenotypes, low bone mass and skeletal fragility with enhanced osteoclast number and/or activity^27–31^. Despite these findings, the exact molecular mechanisms that link SLRPS to osteoclast function are still not clear. Because osteoclasts are not known to express significant levels any SLRP^27^, this cannot be the result of direct effect on [pre]osteoclasts and therefore must be attributed to BMSC/osteoblast/osteocyte cross talk with osteoclast precursors and osteoblastic regulation of osteoclastogenesis.

Previous studies have demonstrated a direct binding between type I SLRPs and TNFα, both through the protein core and, to a lesser extent, the GAG chains^32^. *Bgn* KO mice are hyper responsive to lipopolysaccharide (LPS) induction^33^, and lumican (a type II SLRP) null mice show a higher inflammatory response and high TNFα levels in bacterial keratitis^34^. TNFα has also been shown to enhance the expression of PGs, facilitating remodeling of the ECM as part of the early stages of the inflammation process^35^. Moreover, SLRPs have been shown to regulate osteoclastogenesis via their interaction with both M-CSF and RANKL, as well as with OPG^36–38^, a key inhibitor of osteoclast differentiation and activity^39,40^.

We and others found that mice deficient in one SLRP can compensate with up-regulation of related SLRPs^41,42^. In order to uncover the potentially masked functions of SLRPs due to compensation, we created mice unable to make more than one SLRP.

Here we show that despite what seems like a normal embryonic development, mice lacking both the type I SLRP Bgn along with the type II SLRP Fmod have a strikingly low bone mass. The diminished bone mass is the result of enhanced osteoclast differentiation and activity. We show that the lack of these two SLRPs reduces the ability of osteoblastic cells to retain TNFα and RANKL at the cell surface and the net effect of both over-active cytokine stimulation leads to enhanced osteoclastogenesis. Finally, we show that adding either Bgn or Fmod to preosteoclast cultures reduces their ability to form TRAP positive multinucleated cells, confirming our findings that these two SLRPs are needed to harness osteoclast activity during normal bone homeostasis.

## Results

### BgnFmod KO mice are shorter and thinner than WT and have smaller growth plates

**Figure 1:**
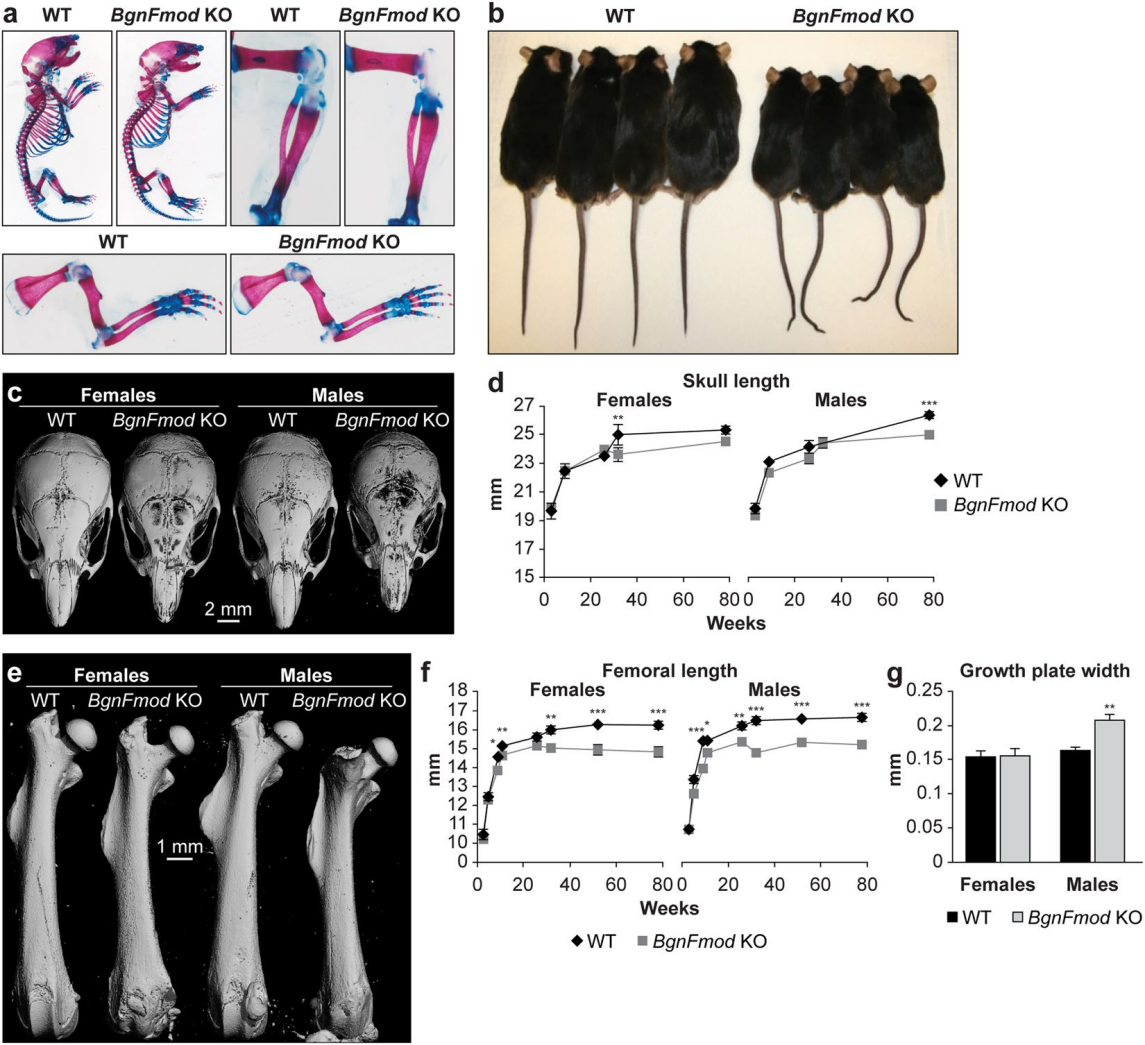
Gross phenotype of BgnFmod KO mice: (**a**) Representative image of alcian blue/alizarin red staining of E17 WT and *BgnFmod* KO mice. Upper left panels: image of whole skeleton, right panels: image of hind limb, lower panels: image of forelimb. (**b**) Representative full body image of female (two left) and male (two right) WT and *BgnFmod* KO mice, showing the size difference and the “kinky” tail of *BgnFmod* KO mice. (**c**) 3D rendering of µCT scans of 5w old skulls showing the difference in skull length as well as the difference in mineralization between *BgnFmod* KO mice and WT mice. Scale = 2 mm. Images are from median samples. (**d**) Quantitative measurement of skull length at different age groups. Data are mean ± SE obtained from N = 5–10 mice per age group. Analyzed by two-way ANOVA. (**e**) 3D rendering of µCT femoral scans from 11w old WT and *BgnFmod* KO mice. Bar = 1 mm. (**f**) Femoral dimensions of WT versus *BgnFmod* KO mice at different time points. Data are mean ± SE obtained from N = 4–10 mice per age group. Analyzed by two-way ANOVA. (**g**) Thickness of distal femoral growth plate of 5w old WT and *BgnFmod* KO mice. Data are mean ± SE obtained from N = 4–5 mice per group, analyzed by unpaired, 2-tailed Student’s T test. *p < 0.05; **p < 0.01; ***p < 0.001.

*BgnFmod* double-deficient mice, generated as previously reported^43^, were backcrossed for 10 generations with C57BL/6J mice to create this genotype with a pure genetic background. The ablation of both genes was routinely verified (Suppl. Figure 1). Both male and female *BgnFmod* KO mice reach full gestation and are viable and normal at birth. Examination of the *BgnFmod* embryos’ skeleton did not reveal any abnormalities in the development of either bone or cartilage compared with WT (Fig. 1a); however, starting at 5 weeks of age, *BgnFmod* KO mice start falling behind WT mice in both gross weight and length, a feature that becomes more exaggerated with age (Fig. 1b–f). µCT analysis of both skull length (a representative of flat bones) (Fig. 1c,d), as well as femur length (a representative of long bones) (Fig. 1e,f) between the ages of 3 and 78 weeks revealed that although *BgnFmod* KO and WT controls started with similar skull and femoral dimensions, by 9 weeks of age the dimensions of both the skulls and femurs were significantly reduced in the *BgnFmod* KO mice compared with those of the WT. While the skulls of the DKO are clearly affected with a low bone mass phenotype, in this paper we focused on the axial skeleton to gain further mechanistic insight. In order to determine whether this growth retardation is a developmental defect, a detailed analysis of the growth plate of 5 week old mice was conducted. Compared with their WT counterparts the male *BgnFmod* KO mice presented thicker growth plates (26.52% increase; Fig. 1g) suggesting that, at least in the males, the lack of SLRP expression in the growth plate may interfere with cartilage maturation and endochondral bone elongation. Another phenotypic feature of the *BgnFmod* KO mice, that becomes more severe with age is a “crooked/kinky tail” (Fig. 1b); however, X-rays of the tails showed no skeletal malformation or distortion (data not shown).

### Morphological changes in BgnFmod KO mice long bones

**Figure 2:**
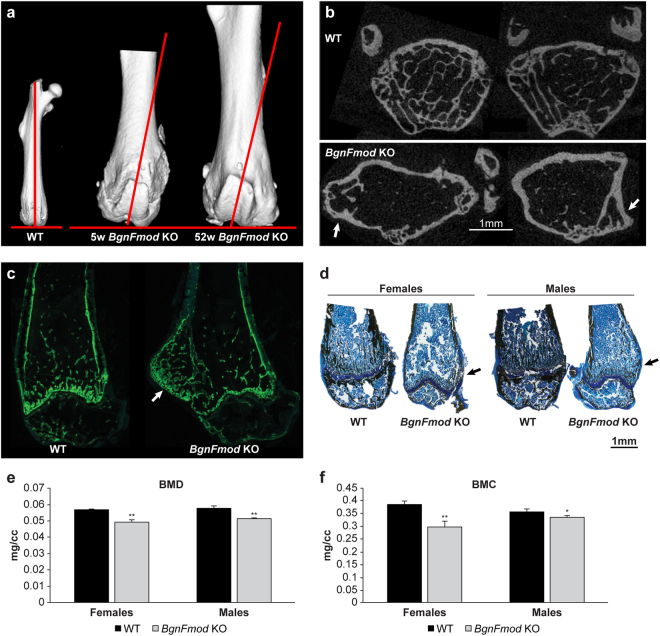
Morphological changes and reduced bone content in the long bones of *BgnFmod* KO mice. (**a**) 3D µCT reconstruction of femora, showing the misalignment of the patellar groove with the long axis of the femoral shaft in the *BgnFmod* KO mice. Images are from median samples. WT femur from 11w old female. (**b**) Representative 2D axial projections of distal metaphysis of 32w old male WT and *BgnFmod* KO mice, showing the changes in morphology as well as the bony protuberance. Bar = 1 mm. (**c**) Representative histological section of calcein double labeling of distal femoral metaphysis of 11w old mice. Arrow pointing at bony lateral projection with dense trabecular architecture in the femur of *BgnFmod* KO mice. (**d**) Von-Kossa staining of 5w old femora presenting bony lateral projection and less mineralized bone in the *BgnFmod* KO mice compared with WT. Bar = 1 mm. (**e**) Bone mineral density (BMD) and (f) bone mineral content (BMC) of 11w old WT and *BgnFmod* KO mice. Obtained by DEXA analysis of whole body. Data are mean ± SE obtained from N = 5–7 mice per group, analyzed by unpaired, 2-tailed Student’s T test. *p < 0.05; **p < 0.01.

In order to assess the importance of Bgn and Fmod on bone structure, we analyzed the skeletal phenotype in greater detail. Using µCT scans, we observed changes in the gross anatomy of the *BgnFmod* KO femoral bone. As early as 5 weeks of age, and worsening as the mice grew older, both male and female *BgnFmod* KO mice exhibited a lateral protuberance in the distal metaphysis of the femur. In addition, the trochlea (patellar groove) was not aligned with the long axis of the femoral shaft. This deviation from the long axis of the bone became more pronounced with time (Fig. 2a). To further investigate the nature of this lateral protuberance, we generated axial projections as well as calcein double labeled and Von-Kossa stained frontal histological sections of the distal metaphysis of the femur (Fig. 2b–d). The axial projections clearly show a distorted anatomical shape of the *BgnFmod* KO femur with a “separated” bony lateral projection, which displays its own, dense trabecular architecture (Fig. 2b–d). The *BgnFmod* KO mice displayed a pronounced reduction in femoral bone mass (Fig. 2d). The Von-Kossa staining also indicated the *BgnFmod* KO mice suffered from low bone mass, a finding that was further confirmed, by DEXA analysis of the whole mice. In both male and female, markedly lower bone mineral density (BMD) and bone mineral content (BMC) levels were demonstrated in the *BgnFmod* KO mice (Fig. 2e-f).

### Skeletal phenotype of BgnFmod KO mice

**Figure 3:**
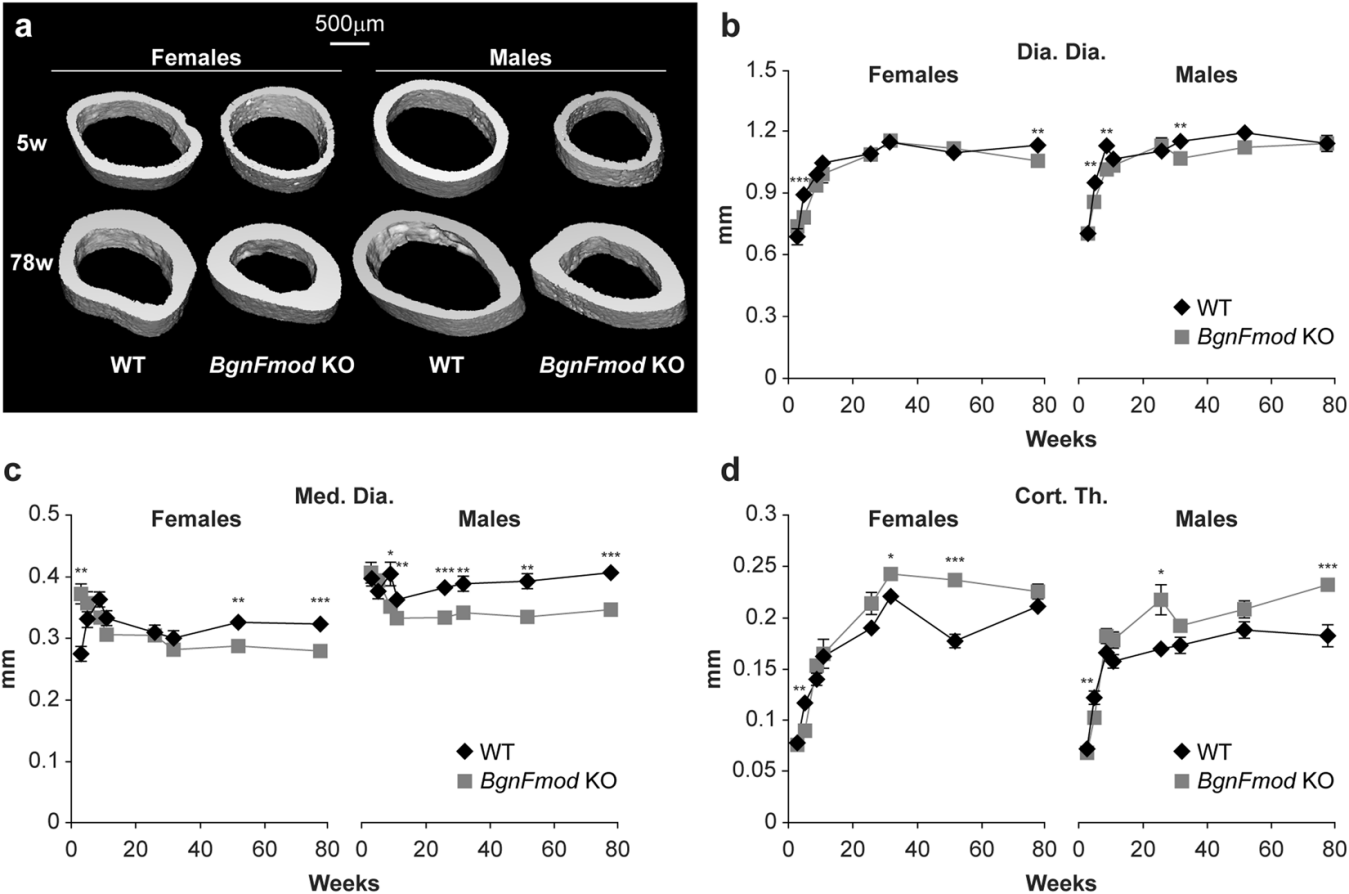
Cortical dimensions of *BgnFmod* KO mice versus WT mice. (**a**) 3D µCT reconstruction of femoral mid-diaphyseal cortical bone at 5w and 78w old. Images from each group were obtained from animals with median cortical thickness bone. Bar = 500 µm. (**b**–**d**) Quantitative µCT analysis of: (**b**) mid-diaphyseal diameter (Dia.Dia.); (**c**) medullary cavity diameter (Med.Dia); (**d**) mid-diaphyseal cortical thickness (Cort.Th.). Data are mean ± SE obtained from N = 4–10 mice per age group. *p < 0.05; **p < 0.01; ***p < 0.001 by two-way ANOVA.

Since the skeleton’s state is influenced by the age of the organism, multiple age groups, representing bone mass accrual stages (3–9 weeks of age), a homeostasis stage (11 weeks of age) and the age-related bone loss stages (26–78 weeks of age) were analyzed. Both the genotype and age of the mice contributed (both as main effects as well as the interaction between them) to the observed skeletal phenotype. µCT analysis of the cortical parameters, measured in a diaphyseal segment extending 1 mm distal from the midpoint between the femoral ends, showed that starting at 9 weeks of age, *BgnFmod* KO mice had substantially thicker cortices than their WT counterparts (Fig. 3a,d). Because, at most time points, there were no significant differences in the diaphyseal diameter between the *BgnFmod* KO and WT mice (Fig. 3b), the increased cortical thickness also resulted in a significantly reduced medullary cavity diameter (Fig. 3c). The increased cortical thickness at the expense of the medullary cavity could be due to increased endocortical bone formation and/or decreased bone resorption. Interestingly, at different ages, the *BgnFmod* KO mice exhibited sexual dimorphism, whereas, at most time points the *BgnFmod* KO male mice had reduced medullary cavity diameters, the female *BgnFmod* KO showed significantly reduced medullary diameter only at an older age (Fig. 3c).

**Figure 4:**
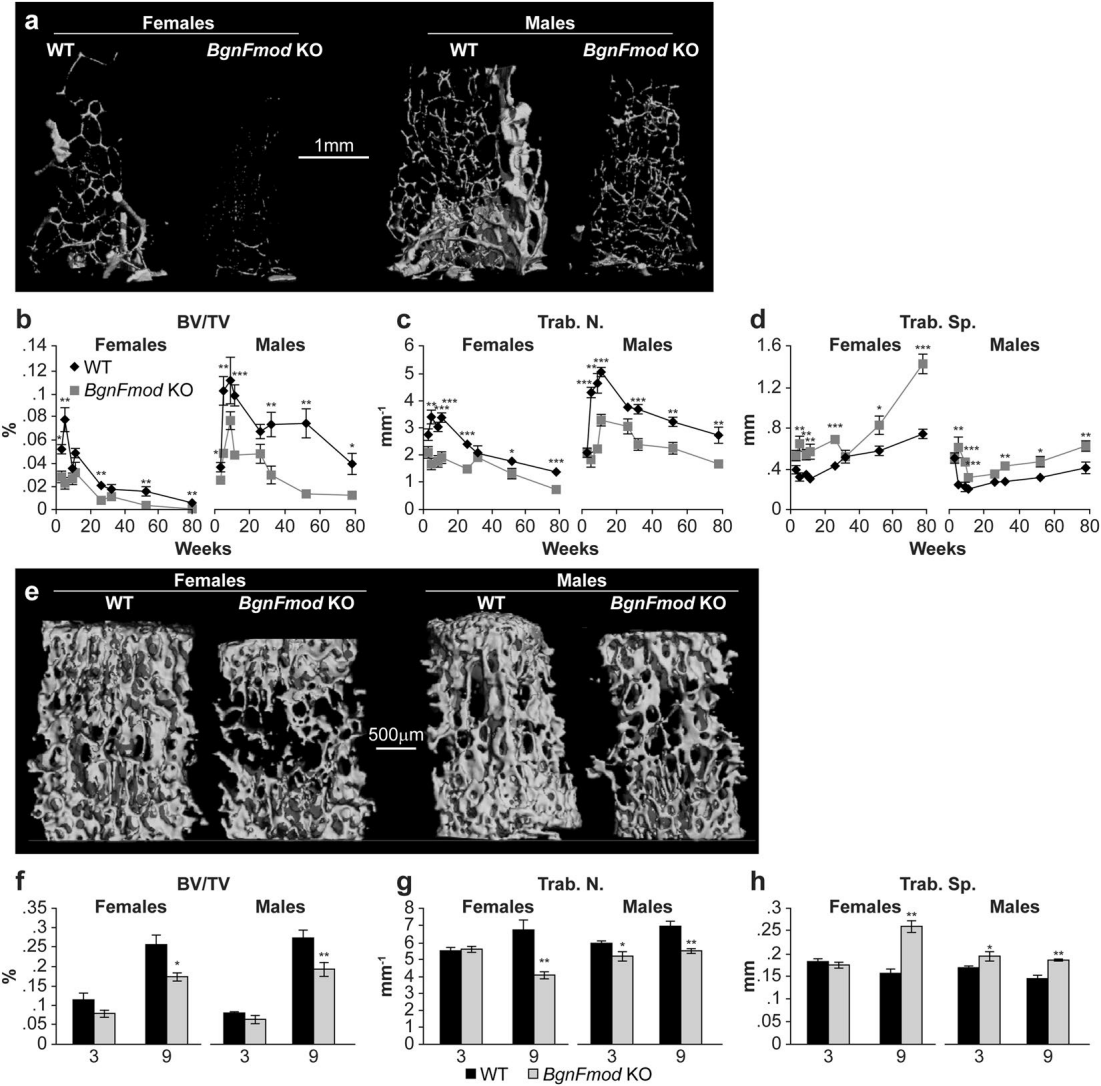
Low trabecular bone mass phenotype in *BgnFmod* KO versus WT mice. (**a**) 3D rendering of distal femoral metaphyseal bone of 52w old mice. Images from each group were obtained from animals with median metaphyseal trabecular bone density values. Bar = 1 mm. (**b**–**d**) Quantitative µCT analysis of: (**b**) Trabecular bone volume density (BV/TV); (**c**) Trabecular number (Trab.N.); (**d**) Trabecular spacing (Trab.Sp.). Data are mean ± SE obtained from N = 4–10 mice per age group. *p < 0.05; **p < 0.01; ***p < 0.001 by two-way ANOVA. Trabecular bone parameters were also analyzed in L3 vertebral bodies: (**e**) 3D µCT rendering of trabecular BV/TV at 9w old WT and *BgnFmod* KO mice. Bar = 500 µm. (**f**–**h**) Quantitative μCT analysis of: (**f**) Trabecular bone volume density (BV/TV); (**g**) Trabecular number (Trab.N.); (**h**) Trabecular spacing (Trab.Sp.). Data are mean ± SE obtained from N = 5 mice per age group. *p < 0.05; **p < 0.01; ***p < 0.001 by unpaired, 2-tailed Student’s T test.

The trabecular bone parameters were analyzed at the secondary spongiosa of the distal femoral metaphysis. Compared with their WT controls, both male and female *BgnFmod* KO mice exhibited strikingly low bone mass (LBM) phenotype (Fig. 4a). Starting at 3 weeks of age and getting worse over time, both male and female *BgnFmod* KO mice had significantly low BV/TV compared with their WT counterparts (Fig. 4b), which was a result of a marked decrease in trabecular number (Fig. 4c), with no major differences in the trabecular thickness (data not shown), resulting in an increase in trabecular spacing (Fig. 4d) and a compromised trabecular architecture. Trabecular bone parameters were also analyzed in the bodies of L3 vertebrae, where similar results of lower BV/TV due to decreased trabecular number were found (Fig. 4e–h).

The observed age dependent bone phenotype is correlated with the level of expression of these two SLRPs in bone. BMSCs extracted from WT animals of different ages as well as whole bone sections of different aged mice showed that, in the natural state, mRNA as well as protein expression levels of both Bgn and Fmod change with age (Figure S2). In young mice the two SLRPs are highly expressed in bone forming cells of all developmental stages: from the chondroblasts/cytes of the growth plates, the chondro-osteoblast transition of the primary spongiosa, by the lining cells surrounding the trabeculi, by periosteal cells as well as by osteocytes already embedded in mineralized matrix (Figures S1C and D and S2). Interestingly, as the mice mature (3 months) and age, the expression levels of both Bgn and Fmod drop significantly and remain relatively low until 78 weeks of age.

### Osteoblasts from BgnFmod KO mice have a higher differentiation potential and increased activity

**Figure 5:**
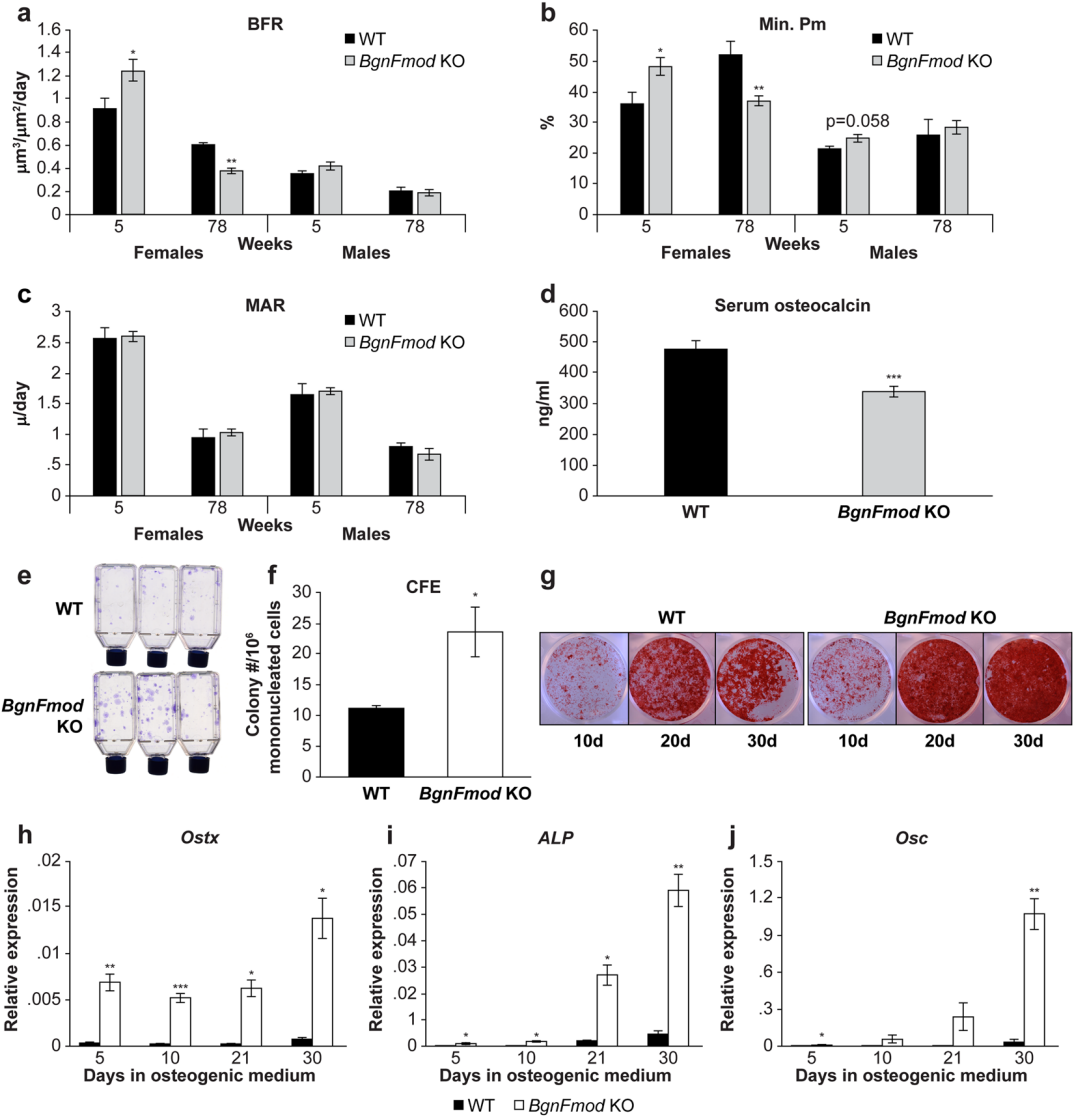
Young *BgnFmod* KO mice have enhanced bone formation and overactive osteoblasts. (**a**–**c**) Dynamic histomorphometric parameters based on fluorescent visualization of calcein fluorochrome in trabecular compartment of distal metaphysis of femur. (**a**) Bone formation rate (BFR); (**b**) mineralizing perimeter (Min.Pm.); (**c**) Mineral appositional Rate (MAR); Data are mean ± SE obtained from N = 4–6 mice per group. (**d**) Osteocalcin serum levels. Serum was obtained from 5 w old mice before euthanization and measured using commercial ELISA kit. (**e**–**j**) *In-vitro* BMSC culture derived from 9 w old WT and BgnFmod KO mice. (**e**) Representative image of colony-forming efficiency showing the number of colonies initiated by a single colony-forming unit-fibroblast formed. (**f**) Enumeration of colonies shown in (**e**). Data are mean ± SE obtained from N = 3 cultures (3 different animals in 3 different cultures) per genotype. (**g**) Alizarin red S staining of BMSCs cultures grown in osteogenic medium for designated periods of time. (**h**–**j**) Quantification of the relative expression levels of osteogenic differentiation markers measured by real-time PCR in mRNA extracted from *BgnFmod* KO and WT BMSCs grown in osteogenic medium for up to 30d. (**h**) Osterix (*Ostx*); (**i**) alkaline phosphatase (*ALP*); (**j**) Osteocalcin (*Osc*). Data are mean ± SE obtained from N = 3 cultures obtained from 3 separate mice per genotype. *p < 0.05; **p < 0.01; by unpaired, 2-tailed Student’s T test.

To gain further insight into the processes leading to the *BgnFmod*-deficiency-induced LBM phenotype, bone formation was analyzed using calcein double labeling (in an age dependent interval). The distal metaphysis of the femur was analyzed by fluorescence visualization. In the trabecular compartment, the female *BgnFmod* KO mice displayed noticeably increased bone formation rate (BFR) at 5-week of age and strikingly reduced BFR at 78-weeks (Fig. 5a). These differences, compared with WT, were mainly due to an increase and later decrease in the mineralization perimeter (Min.Pm.), a surrogate for osteoblast number (Fig. 5b) whereas the mineral appositional rate (MAR), a representative of osteoblast activity, was unaffected (Fig. 5c). Though none of these parameters were statistically significant in the male mice, at 5-weeks of age, the *BgnFmod* KO showed a trend towards increased BFR, similarly due to a slight increase in Min. Pm. with no change in the MAR (Fig. 5a–c). Similar analyses performed when the mice were 11 weeks old did not reveal any significant differences between *BgnFmod* KO and WT mice in either males or females (Fig. S3).

Endocortical labeling revealed similar results. At 5 weeks, female *BgnFmod* KO displayed increased BFR associated with increased Min.Pm. and at 78-weeks the opposite was found- that is, both the endosteal BFR and Min.Pm of the *BgnFmod* KO females were noticeably reduced compared with their WT counterparts (Fig. S4A and B). For the males, only at 78-weeks was the endocortical BFR significantly diminished compared to WT (Fig. S4A and B). For both males and females, the MAR was unaffected (Fig. S4C). Adding another level of complexity, osteocalcin serum levels, obtained from 5-week old mice, were found to be significantly lower in the *BgnFmod* KO (Fig. 5d) animals. Circulating osteocalcin, which is an osteoblast-specific protein expressed by mature osteoblasts and incorporated into the bone matrix, is thought to reflect the portion of newly synthesized protein that does not bind to bone or the matrix incorporated osteocalcin that is being released into the circulation during bone resorption^44^.

Although the gender differences were already apparent from the µCT analysis, the combined results from that and dynamic histomorphometric analysis are somewhat conflicting in light of a LBM phenotype in both male and female *BgnFmod* KO mice from a young age, and might point towards high turnover during the bone accrual period. We next went on to investigate the biological properties and activity of osteogenic cells derived from BMSCs, or calvarial osteoblastic cells.

To assess the *BgnFmod* KO mice’s frequency of osteoprogenitors, the colony forming efficiency (CFE) of BMSCs was tested. Consistent with the increased BFR at a young age, seen by the dynamic histomorphometric analysis, BMSCs derived from 9-week old *BgnFmod* KO showed increased CFE compared with the cells derived from their WT counterparts (Fig. 5e,f). Moreover, when these BMSCs were grown in medium that promotes osteogenic differentiation, the *BgnFmod* KO-derived cells showed an accelerated mineralization rate (Fig. 5g). Similarly, when mRNA was extracted from cells grown for different lengths of time in osteogenesis promoting medium and expression of osterix (*Ostx*), alkaline phosphatase (*ALP*) and osteocalcin (*Osc*), representing early, mid and late osteoblastic differentiation stages respectively, were measured, the expression of all these osteogenic markers was significantly increased in the mRNA derived from *BgnFmod* KO mice (Fig. 5h–j). In fact, even after 30 days in osteogenic medium, the WT-derived cells did not reach 50% of the *BgnFmod* KO-derived cells’ expression of these osteogenic markers. When the mineralization ability of BMSCs derived from young (5-week old) vs. old (78-week old) mice was compared, the “young” *BgnFmod* KO cells showed enhanced mineralization, whereas the “old” *BgnFmod* KO cells were lagging compared to the WT derived cells (Fig. S5A). Taken together these data confirmed the histomorphometric results and indicate that young *BgnFmod* KO osteoblastic cells have, at least *in-vitro*, increased osteoprogenitor number and an enhanced ability to differentiate and mature.

### Effect of BgnFmod depletion on osteoclasts

**Figure 6:**
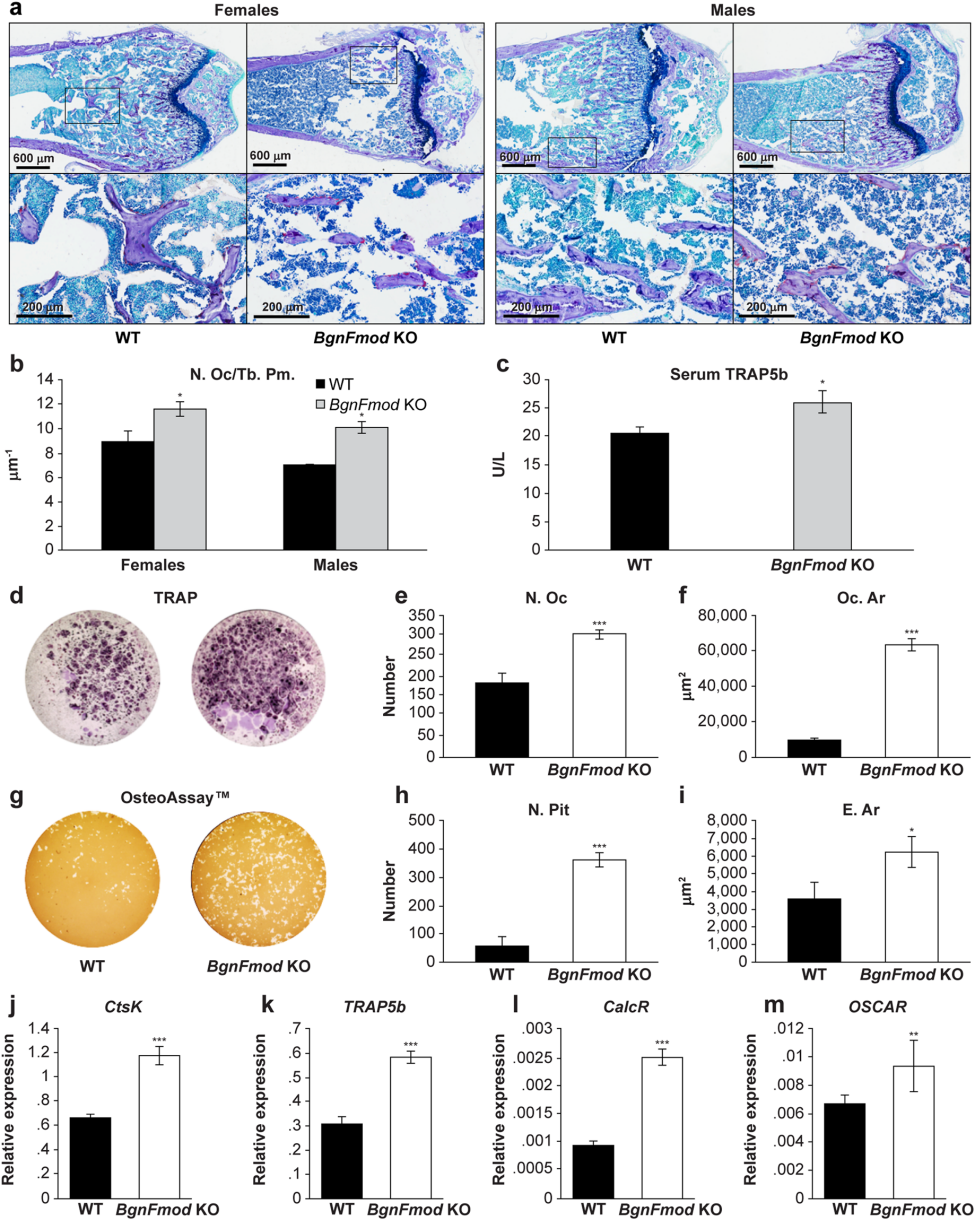
*BgnFmod* deficiency leads to increased osteoclastogenesis and enhanced osteoclast activity. (**a**) Representative images of histological sections of distal femoral metaphysis stained with TRAP. Note increased number of TRAP positive multinucleated osteoclasts in both female and male *BgnFmod* KO mice compared with WT measured at 5 w of age. Upper panel low magnification, bar = 600 µm; Boxed areas are shown in higher power at lower panel, bar = 200 µm. (**b**) Quantitative analysis of osteoclast number per trabecular perimeter (N.Oc/Tb.Pm.). Data are mean ± SE obtained from N = 5 mice per group. *p < 0.05 by unpaired, 2-tailed Student’s T test. (**c**) TRAP5b serum levels. Serum was obtained from 5w old mice before euthanization and measured using commercial ELISA kit. Data are mean ± SE obtained from N = 5 mice. *p < 0.05 by unpaired, 2-tailed Student’s T test.. (**d**–**m**) *In-vitro* osteoclast cultures. (**d**) TRAP staining of osteoclast progenitors derived from WT or *BgnFmod* KO mice bone marrow. (**e**,**f**) Quantification of results shown in (**d**). (**e**) Osteoclast number per well (N.Oc). (**f**) Average size of osteoclast formed (Oc.Ar). (**g**) Pit formation assay of osteoclast precursors plated on osteo-mimetic plates. (**h**,**i**) Quantification of results shown in (**g**). (**h**) Pit number per well (N.Pit). (**i**) Eroded area (E.Ar). For (**d**–**i**) Data are mean ± SE obtained from N = 5 wells per animal 3 separate mice per genotype. *p < 0.05; ***p < 0.001 versus WT by unpaired, 2-tailed Student’s T test. (**j**–**m**) mRNA expression of osteoclastogenic markers in osteoclasts cultures derived from bone marrow. Shown are the relative levels of: (**j**) Cathepsin K (*CtsK*); (**k**) Tartrate-resistant acid phosphatase 5b (*TRAP5b*); (**l**) Calcitonin receptor (*CalcR*); (**m**) Osteoclast-associated immunoglobulin-like receptor (*OSCAR*). Data are mean ± SE obtained from N = 3 wells per animal. ** p<0.01;  ***p < 0.001 by unpaired, 2-tailed Student’s T test.

Because bone mass is a result of a concerted activity of osteoblasts and osteoclasts, and because the osteoblastic side of the bone balance did not explain the LBM phenotype seen in the young *BgnFmod* KO mice, our next step was to closely investigate osteoclastic activity. TRAP stain-based osteoclast counts performed on histological sections from 5-week old mice showed a substantial increase in the number of osteoclasts per trabecular perimeter (Fig. 6a,b) in the *BgnFmod* KO mice compared with their WT controls. Additionally, when the endocortical surfaces of these bone were examined, while not statistically significant (mostly due to very low number of visible osteoclasts), a trend towards decreased number of osteoclast per endosteal perimeter was noticed for both young and old mice in both genders (Fig. S4). A significantly higher level of TRAP5b in the serum of 5-week old *BgnFmod* KO mice was measured (Fig. 6c), indicating a higher number of osteoclasts in these young mice.

When bone marrow cells from both mature and aged *BgnFmod* KO and WT mice were cultured under conditions that promote osteoclastogenesis, there were higher numbers of TRAP positive, multinucleated cells in the cultures derived from the *BgnFmod* KO mice (Figs 6d,e and S5B), and these cells had an average surface area ~6 times larger than the WT-derived cells (Fig. 6f). Furthermore, when similar cells were grown in osteoclastogenesis- promoting conditions in biomimetic coated wells (Fig. 6g), the *BgnFmod* KO-derived osteoclasts were able to produce larger numbers of eroded pits with almost twice the average eroded area (Fig. 6h,i), suggesting that *BgnFmod* KO mice have more osteoclast progenitors, higher osteoclastogenic differentiation ability and increased bone resorption ability and that they retain this ability even at an older age. When mRNA expression of osteoclastogenic markers, such as cathepsin K (*CtsK*), Tartrate-resistant acid phosphatase 5b (*TRAP5b*), Calcitonin receptor (*CalcR*) and Osteoclast-associated immunoglobulin-like receptor (*OSCAR*) were measured, the *BgnFmod* KO-derived cells exhibited significantly higher levels of expression, providing additional evidence of increased osteoclast differentiation ability (Fig. 6j–m). Taken together, the µCT, *in-vivo* histomorphometric and TRAP analysis as well as both osteoblastic and osteoclastic *in-vitro* data indeed point towards a high bone turnover rate in the young *BgnFmod* KO mice and an enhanced bone resorption over bone formation at an older age.

### Bgn and Fmod bind and retain TNFα at the cell surface and BgnFmod KO mice are highly sensitive to TNFα signaling

**Figure 7:**
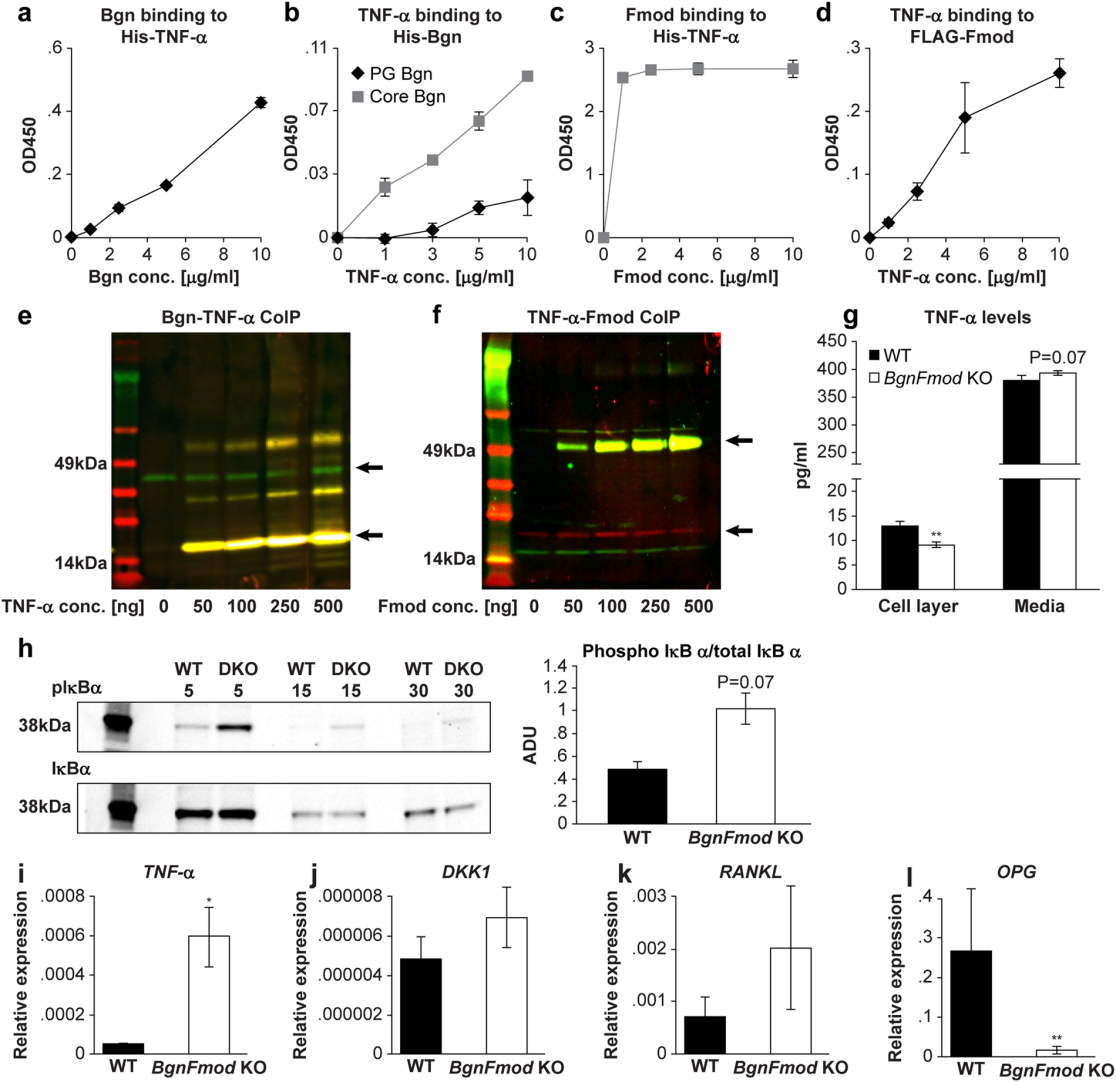
Bgn and Fmod bind TNFα and sequester it at the cell surface. (**a**–**d**) Solid-phase binding assays. (**a**) TNFα was bound to plates and increasing concentration of PG-Bgn added. (**b**) PG-Bgn or Bgn core protein were bound to plates with increasing concentrations of TNFα. (**c**) TNFα was bound to plate and increasing concentrations of Fmod were added. (**d**) Fmod was bound to plate with increasing concentrations of TNFα. Data are mean ± SE obtained from N = 3 wells. (**e**,**f**) CoIP precipitation analysis. (**e**) Bgn core protein was bound to beads and incubated with increasing concentrations of TNFα. Bound protein complexes were eluted and incubated with anti- TNFα antibodies. Yellow bands (17 kDa) are TNFα. Green bands (43 kDa) are Bgn. (**f**) TNFα was bound to beads and incubated with increased concentrations of Fmod. Bound protein complexes were eluted and incubated with anti- Fmod antibodies. Yellow bands at 49 kDa represent Fmod. Red bands (17 kDa) are TNFα. (**g**) TNFα levels measured in cell layer of BMSCs and medium. Data are mean ± SE obtained from N = 3 cultures per animal from 3 different animals per genotype. **p < 0.01 versus WT. (**h**) Left panel: pIκBα and total IκBα expression in BMSCs treated with TNFα. Right panel: Densitometric analysis (Arbitrary densitometric units, ADU) of pIκBα/IκBα expression. Data are mean ± SE obtained from N = 3 cultures derived from 3 separate mice per genotype analyzed by unpaired, 2-tailed Student’s T test. (**i**–**l**) mRNA expression of: (**i**) *TNFα*; (**j**) *DKKI*; both tested in BMSCs. (**k**) *RANKL*; (**l**) *OPG*; both tested in calvarial osteoblastic cells. Data are mean ± SE obtained from N = 3 cultures per animal from 3 different animals per genotype. *p < 0.05; **p < 0.01 by unpaired, 2-tailed Student’s T test.

Osteoclast cells express only negligible levels of Bgn and Fmod (data not shown), and osteoblast derived signals are known to control osteoclast differentiation and activity^40,45–47^. We next investigated ways by which *BgnFmod* depleted osteoblasts might be able to influence osteoclastogenesis. Previous reports showing the ability of TNFα stimulation to promote osteoclastogenesis^8,9^, led us to hypothesize that SLRPs may bind and immobilize TNFα, so that when they are absent, TNFα activation will be altered. We examined whether there is a direct binding between TNFα and either Bgn or Fmod. Using TNFα and either Bgn or Fmod as both prey and bait proteins interchangeably, we showed a dose-response binding ability (Fig. 7a–d). Moreover, when we used only the core Bgn protein and compared its ability to bind TNFα compared with a GAG-chain supplemented form of Bgn, we found that the core protein was actually able to bind the TNFα protein better (Fig. 7b), implying the binding sites are probably located on the core protein itself. Similar dose-dependent interactions between TNFα and both Bgn and Fmod proteins were found when we utilized coimmunoprecipitation (coIP) pull down assays (Fig. 7e,f). These data suggest that the Bgn and Fmod, located in the ECM, might sequester and therefore control the availability of TNFα as an osteoclast differentiation factor. To further explore this possibility, we grew BMSCs in osteogenic medium and measured the levels of TNFα that were shed into the medium, as well as the levels retained by the cells. Lower levels of TNFα were found to be attached to the cell layer of *BgnFmod* KO cells compared with a higher level of bound TNFα found in the WT-derived cells (Fig. 7g). To confirm that *BgnFmod* KO-derived osteoblastic cells are more susceptible/sensitive to TNFα activation, we grew BMSCs with osteogenic promoting medium for 20 days and then treated the cells with active human full length TNFα and measured the levels of the NFκB signal inhibitor, IκBα, and its active phosphorylated form, pIκBα, which is responsible for the proteolytic breakdown of IκBα and the removal of the NFκB cytoplasmic localization restriction. Densitometric analysis of western blotted pIκBα/IκBα corresponding bands, subsequently showed that the *BgnFmod* KO-derived osteoblastic cells were slightly more sensitive to TNFα signaling than the WT-derived osteoblastic cells (Fig. 7h). When the mRNA expression of *TNFα* was tested in BMSCs and calvarial osteoblastic cells, the *BgnFmod* KO-derived cells had noticeably higher levels compared with the WT-derived cells (Fig. 7i). In addition, when we examined the mRNA levels of Dickkopf-related protein 1 (*DKKI*), which is a downstream target of TNFα signaling, we found that the *BgnFmod* KO-derived cells expressed slightly (but non-significantly) higher levels of this WNT signaling pathway inhibitor^48–52^ (Fig. 7j).

### BgnFmod KO osteoblasts express more RANKL but cannot sequester it on the cell surface

Because osteoclastogenesis relies mainly on the presence and availability of RANKL and the ratio between RANKL and its decoy receptor OPG, we decided to examine the expression of these two osteoclast differentiation factors in unchallenged (not-osteogenically promoted) calvarial osteoblastic cells cultures. Compared with their WT counterparts, the *BgnFmod* KO-derived calvarial osteoblastic cells had a marginally higher level of RANKL expression and a significantly lower level of OPG expression (Fig. 7k,l), adding another level of regulation that could control their ability to promote osteoclast differentiation and activity.

**Figure 8:**
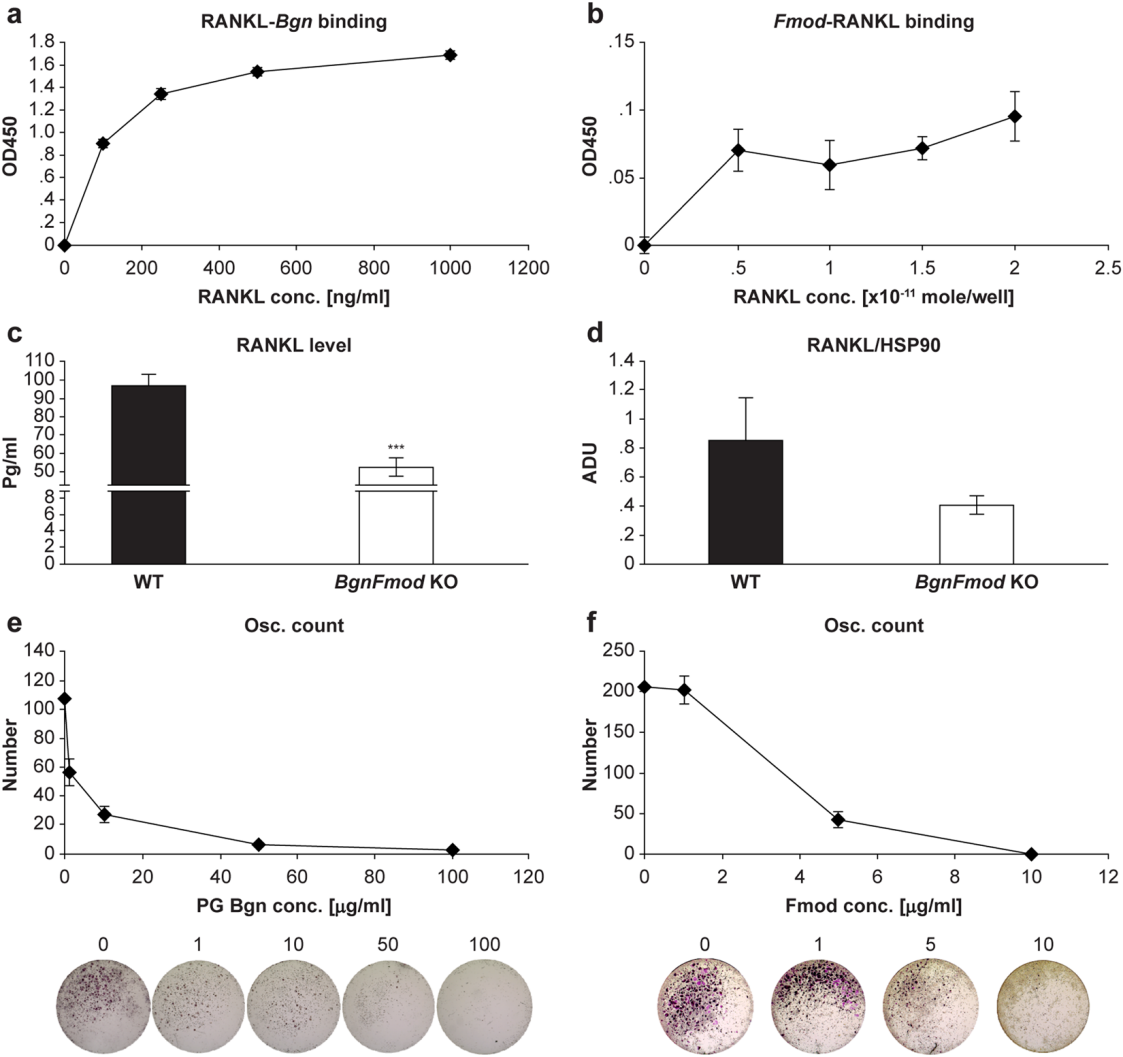
Bgn and Fmod bind and sequester RANKL and can attenuate osteoclastogenesis. (**a**,**b**) Solid-phase binding assays. (**a**) His-tagged PG-Bgn was bound to plate and increasing concentrations of RANKL added. (**b**) C-terminal MYC/DDK Fmod was bound to plate and increasing concentrations of RANKL were added. Data in a & b are mean ± SE obtained from N = 3 wells. (**c**) RANKL levels measured in cell layer of BMSCs grown in osteogenic medium showing that compared with WT *BgnFmod* KO derived cells retain less RANKL at the cell surface. Data are mean ± SE obtained from N = 3 cultures per animal from 3 different animals per genotype. ***p < 0.001 by unpaired, 2-tailed Student’s T test. (**d**) Densitometric analysis of western blot membrane of BMSCs grown in osteogenic medium for 30 days probed against RANKL. Data are mean ± SE obtained from N = 3 cultures per animal from 3 different animals per genotype analyzed using unpaired, 2-tailed Student’s T test. (**e**,**f**) *In-vitro* TRAP staining of osteoclast cultures derived from WT mice grown in the presence of (**e**) PG-Bgn and (**f**) Fmod. Upper panels show enumerations of osteoclast per well, lower panels are representative well images. Data are mean ± SE obtained from N = 5 wells per animal from 3 different animals per genotype.

Although RANKL is synthesized as a transmembrane protein, TACE metalloprotease cleavage of membrane-bound RANKL generates a soluble form of RANKL, which is also bioactive^53^. Previous studies have correlated high levels of soluble RANKL with underlying pathology. To investigate the possibility that the lack of Bgn and Fmod in the ECM also impairs the local restriction of RANKL signaling, allowing it to drift to more remote locations, we again explored whether there is direct binding between RANKL and both Bgn and Fmod. Solid phase binding assays, clearly showed that Bgn binds RANKL in a dose dependent manner (Fig. 8a), whereas although Fmod could also bind RANKL, it was not as linearly directed (Fig. 8b). As with TNFα, we tested the ability of BMSCs to retain RANKL at the cell surface versus the shedding of the active cytokine. When the RANKL levels of BMSCs grown in osteogenic medium were measured using ELISA assays, we found that compared with WT-derived cells, the *BgnFmod* KO-derived osteoblastic cells had strikingly lower levels of RANKL retained at the cell surface (Fig. 8c). In addition, when we conducted a densitometric analysis of the RANKL using western blots of BMSCs grown in osteogenic medium for 30 days, the amount of RANKL extracted from the WT-derived cells exceeded the level of RANKL expression in the *BgnFmod* KO-derived cells (Fig. 8d). Together with the qPCR results indicating the *BgnFmod* KO-derived cells have elevated RANKL gene expression, it is feasible to assume that osteoblastic cells from *BgnFmod* KO mice are less able to retain RANKL on the cell surface and the local cell vicinity. To assess whether Bgn and Fmod has a direct influence on osteoclastogenesis, we added different levels of both proteins to bone marrow derived osteoclast cultures. The addition of either Bgn or Fmod to cells undergoing osteoclastogenesis *in-vitro*, considerably impaired the cells ability to form TRAP positive, multinucleated cells in a dose-dependent manner (Fig. 8e,f).

## Discussion

Despite its inert appearance, the skeleton is actually a very dynamic tissue, undergoing continuous regeneration known as remodeling. In order to maintain the bone’s mass and structural integrity, both crucial to its physiologic functions, the remodeling process is based on the tightly coupled action of osteoclasts and osteoblasts, controlled by complex local (autocrine/paracrine), endocrine and central (neuro-skeletal) regulatory processes^54–56^. With advancing age, this closely synchronized process loses balance as more bone is being resorbed and insufficient new bone is being laid, ultimately leading to age-related bone loss^57–59^.

PGs are major constituents of the ECM that play a crucial role in a myriad of biological processes such as cell attachment, proliferation and differentiation, morphogenesis, tissue repair, inflammation and vascularization^13,14,23–26,60^. They directly bind and interact with a multitude of bioactive molecules such as growth factors, morphogens and receptors, some of which are known regulators of osteoblast number and function^61,62^. The direct but transient association with either the core protein or the GAG chains of SLRPs provides low affinity storage for these molecules and directly controls normal and pathological processes. The presence or absence of SLRPs at specific phases of bone development, the remodeling cycle or fracture healing, can therefore regulate the rate and progression of these processes by means of permitting or restricting diffusion of regulatory molecules^63^.

Bgn and Fmod are members of the SRLP family that are abundant in mineralized tissues. The precise physiologic function of *Bgn* and *Fmod* during embryonal skeletal development has yet to be determined and indeed, analyzing the skeletal phenotype of *BgnFmod* KO deficient mice did not reveal any skeletal developmental problem, however from a very young age significant deficits in bone size, structure and architecture were noticed in the *BgnFmod* deficient mice. The intense expression of Bgn and, to a lesser extent, Fmod at the chondro-osseous junction of the growth plate may explain the changes in bone length and growth plate width demonstrated in this report as a response to the depletion of these two SLRPs. This decrease in femoral length and, at least in the males, the accompanying increase in growth plate thickness are reminiscent of the growth impairment observed in association with other PG deficits^64,65^. Previous investigations measuring the levels of SLRPs made by human osteoblasts taken from donors that ranged from birth to 60 years of age, showed that the production of several small proteoglycans, including biglycan, gradually increases from birth until about 12 years of age, after which there is a gradual but rapid decline until age 60^66^. Though our measurements of normal Bgn and Fmod expression resembles the human data (e.g. high expression at a young age with markedly declining levels as the mice age), the skeletal phenotype of the *BgnFmod* KO mice only kept deteriorating as the mice aged. Taken together these data suggest there is important role for SLRPS, such as Bgn and Fmod, at early stages of bone accrual, and that even at later ages, when the natural levels of these proteins decrease, these shortfalls cannot be overcome. Moreover, as these SLRPs are deposited into the ECM, where they serve, among other functions, as a reservoir for growth factors and cytokines^13,21,22^, any shortcoming in the initial expression, might have a long lasting effect on physiological as well as pathological processes.

We previously reported that *BgnFmod* KO mice suffer from weak and structurally deformed tendons which undergo severe age-dependent ectopic ossification. The tendons’ laxity leads to unstable joints and as a result these mice succumb to early onset osteoarthritis^43,67,68^. The morphological changes we observed in the long, weight-bearing bones of these animals, may be the result of the unstable joints exerting misaligned shear forces on the femurs. On the other hand, these skeletal deformities, already apparent in the young skeleton, might be a co-founding factor leading to unstable joint articulation which promotes joint attrition. In this context, it is interesting to note that loss of function mutations in the human *Bgn* gene were recently identified as one seminal cause leading to joint laxity, abnormally shaped bones and reduced bone growth^41,69^.

The DEXA analysis, together with our thorough longitudinal µCT evaluation of the *BgnFmod* KO mice clearly proves that these SLRPs regulate bone remodeling in both the cortical and trabecular compartments. Though the cortical thickness of *BgnFmod* KO mice is actually increased with age, mostly at the expense of the medullary cavity, the overall phenotype is one of LBM, presenting reduced trabecular number with impaired trabecular architecture represented by decreased connectivity (not shown), especially as these animals age. Moreover, we show the LBM is apparently the result of high turnover at the bone accrual stage, as both the osteoclastic and osteoblastic functions are elevated in the young *BgnFmod* KO, and an “ameliorated” age-dependent bone loss at older ages, as osteoblast number and function diminish but osteoclast over activity (at least theoretically, based on the *in-vitro* data) persists. Our *in-vitro* experiments further demonstrated that the Bgn and Fmod influence on bone cells is both on osteoblasts and then indirectly through them on osteoclasts. Despite the fact that for both male and female *BgnFmod* KO mice, the net skeletal phenotype was that of low bone mass, the exhibited sexual dimorphism in specific skeletal parameters is a common, yet puzzling issue and may be secondary to the basic skeletal sexual dimorphism^70,71^ and/or the differential effects of Bgn and Fmod and their ablation on these basic skeletal sexual dimorphism. Given that the *BgnFmod* KO mice have low bone mass starting at a very young age (5 weeks which is prior to sexual maturation in mice) it is likely that sex hormones are not a primary determinate of the phenotype. It is possible that additional experiments might reveal that male or female *BgnFmod* KO mice will be differentially sensitive to sex hormone depletion by ovariectomy or orchiectomy but further insight into sexual dipmorphism will require additional experiments beyond the scope of this manuscript.

RANKL, which works through the type I transmembrane receptor of the TNFα family RANK^7^, is considered the main osteoclastogenesis promoting cytokine and induces osteoclast differentiation, function and survival by activating both the canonical and non-canonical pathways of NF-κB. Previous studies have shown that TNFα can induce differentiation and survival of osteoclasts in a RANKL independent manner^8,9^. In addition to its direct activation of the NFκB canonical signaling pathway in preosteoclasts^72,73^, causing induction of NFATc1, the master transcription factor of osteoclastogenesis^74,75^, TNFα signaling also promotes the expression of RANKL in BMSCs as well as the expression of RANK by myeloid progenitors, priming them to respond to RANKL^10–12^. Furthermore, TNFα also indirectly regulates osteoclast function. TNFα induces the expression of *DKK1*, an inhibitor of the well-established skeletal signaling pathway wnt^48–52^, thereby interfering with wnt regulated bone formation. Newly activated osteoblasts act as a signal to cease osteoclast activity in the specific foci. The TNFα induced expression of *DKK1* signaling, which blocks osteoblast differentiation, further biases towards imbalanced/unopposed resorption. In this study, though *BgnFmod* KO-derived BMSCs grown in osteogenic medium expressed somewhat higher levels of *DKK1* compared with WT-derived BMSC, the fact that the dynamic histomorphometry at the younger age did not show reduced BFR in the *BgnFmod* KO may indicate that an alternative, non-canonical pathway is responsible for the increased osteoblast function found in this situation.

Although both membrane and soluble forms of TNFα and RANKL are biologically active, the membrane-bound form is the one used under normal conditions, while the soluble form is associated with pathology^76–79^. It is well established that chronic immune activation has a substantial role in bone lysis and loss of bone mass (e.g. osteoarthritis, lupus and osteolytic metastatic reaction)^52,80,81^. One such example is ankylosing spondylitis (AS), which is a chronic inflammatory arthritis, where there is simultaneous destruction and formation of bone and where TNFα has a role in the pathogenesis^82,83^. Some of the symptoms of this disease are stiffness and fusion of joints (especially in the vertebrae) and syndesmophyes (a bony growth inside a ligament), features previously reported in the *BgnFmod* KO mice^43,67,68^. The present study provides the first direct evidence of TNFα involvement that might explain the pathogenesis leading to these phenotypes.

Our finding that both Bgn and Fmod directly bind to TNFα and RANKL, that *BgnFmod* KO mice can retain less TNFα and RANKL in the cell layer of BMSCs, causing over activated NFκB signaling, and even more so the ability of soluble Bgn and Fmod to disturb osteoclast differentiation, even in the presence of M-CSF and RANKL, makes them a new and attractive target for drug development. As opposed to current FDA approved anti-osteoporotic drugs, these two SLRPs could, potentially, act to regulate resorption as well as formation, leading to appropriate rates of bone turnover and, ultimately higher bone mass. In this context, it is important to highlight that Fmod was recently found to induce bone regeneration by reprograming multipotent fibroblasts without genomic alteration or stimulation of tumorgenesis^84,85^. As the world’s population steadily continues to age, osteoporosis and osteoporotic fractures will constitute a growing public health burden. Therefore, a better understanding of the cellular mechanisms of bone remodeling and the effects of different factors on these mechanisms, is likely to identify new targets for therapy that will assist in preventing and reversing bone fragility. Taken together, our data suggests that SLRPs may provide an attractive novel target in the diagnosis and treatment of bone diseases.

## Materials and Methods

### Animals

Mice deficient in both *Bgn* and *Fmod* were generated as previously described^67^. These mice were backcrossed with C57BL/6J mice (Taconic Biosciences) for 10 generations to produce *BgnFmod* KO mice on a pure C57BL/6J background. Mice were bred and kept at the NIDCR/NIH/DHHS animal facility with water and food ad libitum. All animal experiments were approved by NIDCR/NIH/DHHS ACUC. Groups of 4–10 mice, aged 3–78 weeks, were used in each experiment. 17d embryos were used for cartilage-GAG and bone staining. The loss of Bgn and Fmod expression, both at the mRNA and protein levels, was confirmed by RT-PCR (genotyping), qRT-PCR and immunohistochemistry, described in more detail below.

### Cell cultures

Primary bone marrow stromal cells (BMSCs) were derived from the femoral and tibial diaphyseal medullary cavity of *BgnFmod* KO and WT mice. Calvarial osteoblastic cells were obtained from 4–5 day-old mice by successive 1 mg/ml Collagenase-P trypsin (Roche Diagnostics, Sigma Aldrich; respectively) digestions. Cells were grown in αMEM (Gibco) supplemented with 20% lot-selected non heat inactivated FBS (Gemini Bio Products), 100U/ml each of penicillin streptomycin (Gibco), 2 mM/ml glutamax, 0.011 µM/ml, 2-mercaptoethanol (Gibco), 10^−8^ M/ml dexamethasone (termed maintenance medium). To induce osteogenic differentiation, 100 µM/ml L-ascorbic acid phosphate and 2 mM/ml β-glycerophosphate were added to the growth medium. For macrophage-free cultures, BMSCs were expanded up to 2 passages and then CD45-, CD11b- cells were selected using the EasySep kit™ (STEMCELL Technologies) according to manufacturer’s instruction. To assess mineral (Ca^++^) deposition, the cells were washed with PBS, fixed in 95% ethanol and stained for 5 min with a saturated solution of alizarin red S (Sigma-Aldrich).

Primary osteoclast cultures were prepared from the femora and tibiae of *BgnFmod* KO and WT mice and grown for 3 to 5d in medium containing M-CSF and RANKL (R&D Systems) as reported previously^86^. Osteoclast formation was confirmed by osteoclast-specific tartrate-resistant acid phosphatase (TRAP) staining (Sigma- Aldrich). For pit formation analysis, bone marrow-derived monocytes were seeded in Osteoassay™ plates (Corning) and grown in medium containing M-CSF and RANKL (416-ML & 462-TEC respectively, R&D Systems) for 4 days. To allow examination of lacunar resorption, the wells were washed with 10% sodiumhypochloride and observed under light microscopy. Quantitation analysis for both assays was done using ImagePro Analyzer 7.0 (Media Cybernetics, Inc.).

### Embryo staining

E17 mice were stained with a combination of Alcian Blue and Alizarin Red according to established protocols. Briefly, embryos were skinned and fat was removed, they were then fixed in 95% ethanol for 4 days and acetone for another 1 day. Thereafter the embryos were transferred to a solution of 0.1% alcian blue in 70% ethanol, 0.3% alizarin red in 95% ethanol, acetic acid and 70% ethanol for 2–3 days. After extensive washing with both H_2_O and 1% KOH for 12–24 hours, the embryos were processed with successive 24 hour washes with increasing glycerin concentrations^87^.

### Dual-energy X-ray Absorptiometry (DEXA)

At the time of sacrifice mice were weighed and scanned with a DEXA machine (Lunar PIXImus densitometer, GE Healthcare) to determine the total bone mineral density (BMD) and total bone mineral content (BMC). When analyzing these parameters, the skulls were excluded from the region of interest.

### Micro-computed tomography (µCT)

The skeletal phenotype of *BgnFmod* KO mice was first analyzed in whole femurs and L3 lumbar vertebrae using the µCT50 system (Scanco Medical AG, Brüttisellen, Switzerland) at a 10-μm resolution. Mineralized tissues were segmented by a global thresholding software. Standardized nomenclature was used for the bone parameters measured. For the femurs, trabecular parameters were measured at the secondary spongiosa of the distal metaphysis and cortical parameters were determined in a 1mm ring at the mid-diaphyseal region according to previously published guidelines^88^. For the 3^rd^ lumbar vertebra only trabecular parameters were measured. Growth plate thickness was measured in the distal epiphysis of 5-week old mice. Briefly, for each sample, 2D projections from anterior, posterior and mid-longitudinal locations were obtained, the average thickness of the growth plate obtained from the three projections was quantified using the ImagePro Analyzer 7.0 software (Media Cybernetics, Inc.).

### Quantitative RT- PCR

Total RNA was extracted from the cells, purified and reverse-transcribed using RNeasy (Qiagen) and iScript cDNA synthesis kit (170–8891, Bio-Rad) respectively following manufacturers’ instructions. qRT-PCR was performed using iQ SYBR Green Supermix (170–8886, Bio-Rad). Target genes were normalized to S29 and relative expression data was calculated using the ΔΔCt method. A list of primers used is found in Table S1.

### Protein extraction and western blotting

Subconfluent cultures of BMSCs grown in “osteogenic medium” for 20–30 d were serum-starved for 2 hours in 0.5% BSA containing αMEM. To assess TNFα downstream activation, similarly grown cells were incubated for various time periods, ranging from 5 to 30 min, with 10ng/ml of active human full-length TNFα (192134, Abcam) in the same medium. Thereafter, the cells were rinsed twice with ice cold TBS ×1 and whole cell lysates were extracted using M-PER lysis buffer (78501, Thermo Fisher Scientific) supplemented with Complete^®^ protease inhibitor mixture and PhosphoSTOP^™^ (11873580001 and 04906837001, respectively; Roche Diagnostics) and later sonicated and clarified by centrifugation at 12,000 g for 15 min. 40–50 µg of protein, were fractionated using the NuPAGE system (Invitrogen) and then electroblotted onto nitrocellulose membranes. Membranes were blocked and then incubated overnight at 4 °C with IκBα or pIκBα (1:500 dilution of mouse monoclonal 4814& 9246 respectively; Cell signaling) or RANKL (1:200 dilution, goat polyclonal AF462, R&D), HSP90 (1:1000 dilution, rabbit polyclonal sc-7947 santa cruz) was used as housekeeping protein to standardize samples. IRDye^®^ Species specific infrared secondary antibodies (1:10,000 dilution, Odyssey CLx Infrared Imaging System) together with the LI-COR imaging system and Odyssey 2.1 software (LI-COR Biosciences, Lincoln, NE) were used to analyze results.

### Dynamic histomorphometry

Prior to sacrifice WT and *BgnFmod* KO mice were injected with a calcein fluorochrome (Sigma-Aldrich, St. Louis, MO), 15 mg/Kg intraperitoneally. 5-week old mice were injected with calcein 4 days and 1 day prior to sacrifice while 78-week old mice were injected with calcein 12 days and 1 day prior to sacrifice. Following µCT scanning the femurs were embedded undecalcified in methyl methacrylate. Mid-frontal longitudinal sections were prepared. For dynamic histomorphometry based on the vital calcein double labeling the sections were left unstained. To identify osteoclasts, consecutive sections were stained for TRAP (294–67001, Wako Pure Chemical Industries, Osaka, Japan). Quantitation analysis for both assays was performed using ImagePro Analyzer 7.0 (Media Cybernetics, Inc.). The bone formation and resorption parameters were determined in the secondary spongiosa of the distal metaphysis as reported previously^89^ according to a standardized nomenclature^90^.

### Colony-forming Efficiency Assay

To compare the clonogenic potential of primary BMSCs derived from *BgnFmod* KO and WT mice, bone marrow was flushed from femora and tibiae of 9-week old mice, plated in 25 cm^2^ flasks (10^6^ cell/flask) with growth medium and left to grow untouched for 10d. Cells were then fixed with 100% methanol and following washes with PBS were stained with saturated methyl violet (Gibco) for 30 min. The number of colonies was obtained using light microscopy. Only colonies with more than 30 cells were counted.

### Histology and immunohistochemistry (IHC)

Paraffin-embedded decalcified longitudinal sections of either 3- or 11-week old femurs were deparaffinized and hydrated. Von kossa staining was performed as described previously^91^. For IHC, deparaffinized and hydrated sections were incubated for 1 h at 37 °C with ABCase for Bgn or keratanase for Fmod (Seikagaku biobusiness corp.; Japan), following antigen retrieval (Unitrieve, Innovex), and quenching of endogenous peroxidase activity with dual endogenous enzyme block (Dako), sections were blocked with 10% normal goat serum for 1 h at 37 °C. Bgn rabbit antisera, LF-159 (1:500), or Fmod rabbit antisera LF-150 (1:500; both from Dr. Larry W. Fisher, NIH) [Figure S1] were added to sections and incubated overnight at 4 °C. The samples were then incubated with Super PicTure™ Polymer detection kit (Invitrogen) for 10 min at room temperature and detected with ImmPACT™ AEC (Vector laboratories). Mayer’s hematoxylin was used as counterstain. Slides were scanned using an Aperio ScanScope slide scanner.

### Co-immunopercipitation (coIP)

His•Bind^®^ Resin beads (EMD Millipore) were precharged with Nickel sulfate solution. Different concentrations of prey protein were incubated for 5 h at RT on rotating shaker with a constant concentration of His-tagged bait protein (Table S2). The protein mix was allowed to bind to the beads at 4 °C overnight. The next day the beads were spun down, and flow-through collected. Following repeated washes, the beads were resuspended in 10% 2-mercaptoethanol supplemented SDS loading buffer and boiled at 95 °C for 10 min to elute the bound protein. The samples were loaded onto SDS-PAGE gels and then electroblotted onto nitrocellulose membranes. To detect coIP, Primary Antibodies (Table S3; 1:1000 dilutions) against the prey protein were used followed by species specific secondary antibody (LI-COR Biosciences).

### Solid-phase binding assay

Bait proteins were bound to either Pierce^®^ Nickel coated plates (15242; Thermo scientific) or ANTI-FLAG^®^ high sensitivity, M2 coated plates (P2983, Sigma) by 2 h incubation at 37 °C. Unbound protein was removed by repeated washing and non-specific binding sites were blocked with 0.1–1% BSA/TBS. Bait-coated plates were incubated with prey proteins at 4 °C overnight. Binding of the prey protein to the coated plate was detected using primary antibodies raised against the prey proteins, combined with species matched HRP-conjugated secondary antibodies. Plates were read at 450 nm after HRP chromogenic substrate reaction using the TMB-peroxidase substrate system (KPL, Gaithersburg, MD, USA) according to manufacturer’s protocol. List of proteins and primary antibodies used can be found in Tables S2 and S3.

### ELISA

Following 20–30 d growth in “osteogenic medium” BMSCs were washed with PBS twice and incubated in serum-free medium overnight. The following morning, culture medium was collected and concentrated using Amicon^®^ Ultra-4 centrifugal filter units (EMD Millipore). The cells were then washed twice with TBSx1 and whole cell extracts were prepared using 150 µl of M-PER with cOmplete™ EDTA-free protease inhibitor cocktail (Roche Life Science) and PhosSTOP™ phosphatase inhibitor cocktail (Roche Life Science) and later sonicated as described for western blotting. For measurement of TNFα, after the overnight incubation in serum free medium, the cells were treated with 500 pg/ml of active human full-length TNFα (Abcam 192134) in 1% BSA containing αMEM for 60 min and the medium and whole cell lysate were then collected. Levels of TNFα and RANKL in medium vs. cell layer were measured using Quantikine ELISA Kits (SMTA00B & MTR00 respectively, R&D) according to manufacturer’s instruction.

For serum markers of bone remodeling whole blood was collected retro-orbitally at the time of animal sacrifice. Serum was separated by centrifugation after allowing 2 hour clotting. Serum osteocalcin and TRAP5b were analyzed in the same specimens using commercial EIA kits according to manufacturer’s instructions (osteocalcin: BT-470; Alfa Aesar; TRAP5b: SB-TR103, Immunodiagnostic System Inc.).

### Statistical Analysis

Differences were examined by two tailed Student t-test for comparing two groups and by either one-way or two-way analysis of variance (ANOVA) test for comparing multiple groups. When significant differences were indicated by ANOVA, group means were compared to establish the source of the differences. P < 0.05 was considered statistically significant.

### Study approval

All animal studies were performed in accordance with NIH guidelines under institutionally approved protocols.

## Electronic supplementary material

supplementary information
