# Supplementary material for: Small leucine rich proteoglycans, a novel link to osteoclastogenesis
Source: Sci Rep. 2017 Oct 3;7:12627. doi: 10.1038/s41598-017-12651-6 (PMC5626712; doi:10.1038/s41598-017-12651-6)
Supplement: Supplementary file 1 — supplementary information [file 41598_2017_12651_MOESM1_ESM.pdf]

Supporting Online Material for

Small leucine rich proteoglycans, a novel link to osteoclastogenesis

Kram, Vardit[1]; Kilts, Tina M.[1]; Bhattacharyya, Nisan[2]; Li Li[1] and Young, Marian F[1].

1. Craniofacial and Skeletal Diseases Branch, National Institute of Dental and Craniofacial Research, National Institutes of Health, Bethesda, MD 20892, USA.
2. Scientific Review Branch, Division of Extramural Activities, National Institute of Dental and Craniofacial Research, National Institutes of Health, Bethesda, MD 20892, USA.

Correspondence to Marian F. Young: MSC 4320, Building 30 Room 211, 9000 Rockville Pike, Bethesda, MD 20892-4320.

Phone: 301-496-8860. Email address: myoung@dir.nidcr.nih.gov

Table S1: Primer list

Gene	GenBank^{TM} accession no.	Sense sequence	Anti-sense sequence
S29	NM_009093	5’- GGAGTCACCCACGGAAGTTCGG-3’	5’-GGAAGCACTGGCGGCACATG-3’
Bgn	NM_007542.5	5’-AGACAAACCGACAGCCTGACAAC-3’	5’-GCCAGCAGCAAGGTGAGTAGC-3’
Fmod	NM_021355.3	5’-CCTGATCCTACTAGACCTGAG-3’	5’- GACGGTGTAGACATTGTTGT -3’
Ostx	NM_130458.3	5’-GCTCGTCTGACTGCCTGCCTAG -3’	5’- GTGAGATGCCTGCGTGGATGC-3’
ALP	NM_007431	5’-GCAAGGACATCGCATATCA-3’	5’-CAGTTCGTATTCCACATCAGT-3’
Osc	NM_031368.5	5’-AGACCGCCTACAAACGCATCTATG-3’	5’-GCTGCTGTGACATCCATACTTGC-3’
CtsK	NM_007802	5’-TGGCTCGGAATAAGAACAAC-3’	5’-AAGGAAGGAATCTGAGAAGAGA-3’
TRAP5b	NM_001102404	5’-TGCGACATCAACGAAAGG-3’	5’-TTCCAGAGGCTTCCACAT-3’
CalcR	NM_001042725	5’-AACCATTATCCACGCCATC-3’	5’-GAAGAAGTTGACCACCAGAG-3’
OSCAR	NM_175632	5’-ACTCCACCAGATACTCATTCT-3’	5’-AGTCACTAACATTAGCTGAACAT-3’
TNFα	NM_013693	5’-AGGACTCAAATGGGCTTTC-3’	5’-AGGTCTGAAGGTAGGAAGG-3’
DKK1	NM_010051.3	5’-CGGGCTCTGCTGTCAGTGTGG-3’	5’-AGGGTAGGGCTGGTAGTTGTCAAG-3’
RANKL	NM_011613.3	5’-GTCACTCTGTCCTCTTGGTA-3’	5’-CGCTTCCCGATGTTTCAT-3’
OPG	NM_008764	5’-CCTTGCCCTGACCACTCTTATACG-3’	5’-CCTTCCTCACACTCACACACTCG-3’

Table S2: Protein list

**Protein name**	**Provider**	**Application used**
His-tagged Bgn	151798; Abcam	Solid phase binding assay; CoIP;
His-tagged core and PG-Bgn	Dr. Rick T Owen; Lifecell Corporation	Solid phase binding assay; CoIP; osteoclast culture
C-terminal DKK-tagged recombinant human Fmod	TP306534; Origene	Solid phase binding assay; CoIP; Osteoclast culture
Recombinant human His-tagged TNFα	cyt-494; ProSpec	Solid phase binding assay; CoIP
Human full length TNFα	186083; Abcam	Solid phase binding assay; CoIP
Recombinant mouse TNFα	410-MT; R&D	Solid phase binding assay;
Active human full length TNFα	192134; Abcam	ELISA; WB
Recombinant Mouse RANKL	462-TEC; R&D	Solid phase binding assay; Osteoclast culture

Table S3: Antibody list

**Ab name**	**Provider**	**Application used**
Rabbit anti sera against Bgn	LF-112& LF-159; Dr. Larry Fisher NIH	IHC; Solid phase binding assay;
Rabbit polyclonal against Bgn	49701 & 94460; Abcam	CoIP
Rabbit anti sera against Fmod	LF-149& LF-150; Dr. Larry Fisher NIH	IHC; Solid phase binding assay; CoIP
Rabbit polyclonal against Fmod	81443; Abcam	CoIP
Rabbit polyclonal against TNFα	9739; Abcam	Solid phase binding assay; CoIP
Rabbit polyclonal against TNFα	3707; Cell signaling	Solid phase binding assay; CoIP
Goat polyclonal against RANKL	AF462; R&D	Solid phase binding assay; WB
Mouse monoclonal ANTI-FLAG® M2	F1804; Sigma	Solid phase binding assay
Mouse monoclonal against IκBα	4814; Cell signaling	WB
Mouse monoclonal against pIκBα	9246; Cell signaling	WB
Rabbit polyclonal against HSP90	sc-7947; Santa cruz	WB
Rabbit polyclonal against β-actin	8457; Cell signaling	WB

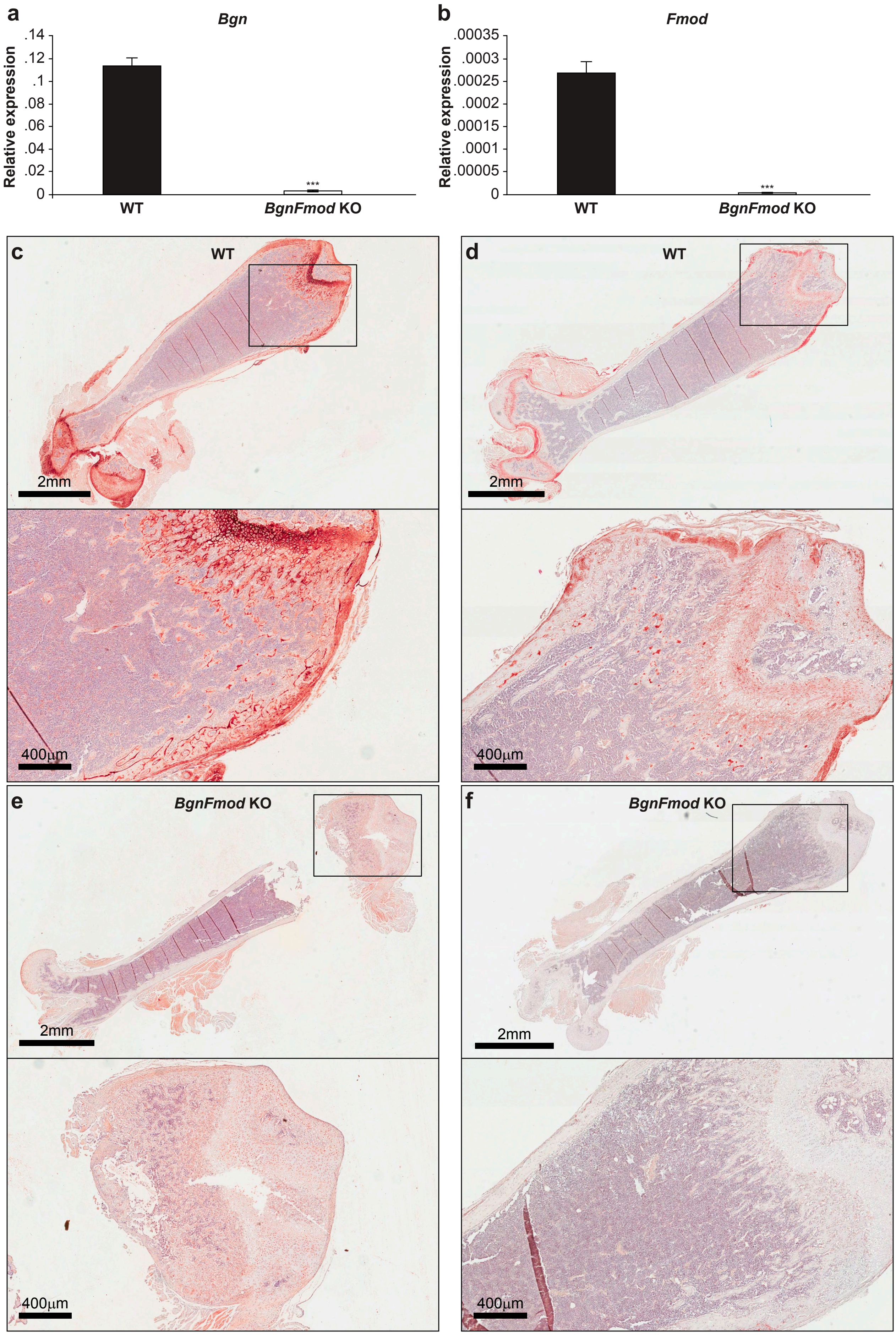

Supplementary Figure 1. Bgn and Fmod expression in bone. (a-b). mRNA expression in BMSCs derived from WT and *BgnFmod* KO mice. A. *Bgn*; B. *Fmod*. Data are mean±SE obtained from N=3 cultures per animal from 3 different animals per genotype. ***, $p<0.001$ compared with WT. (c-f) Representative sections of femurs immunohistochemically probed with: C+E. Bgn; D+F: Fmod. Upper panels low magnification, bar=2 mm; Boxed areas are shown in higher power at lower panels, bar=400µm. Note in WT sections positive staining of both Bgn and Fmod in chondroblasts/cytes at the growth plate, the chondro-osteoblast transition of the primary spongiosa, lining cells surrounding the trabeculi, the periosteal cells as well as by osteocytes already embedded in mineralized matrix chondroblasts/cytes of the growth plates.

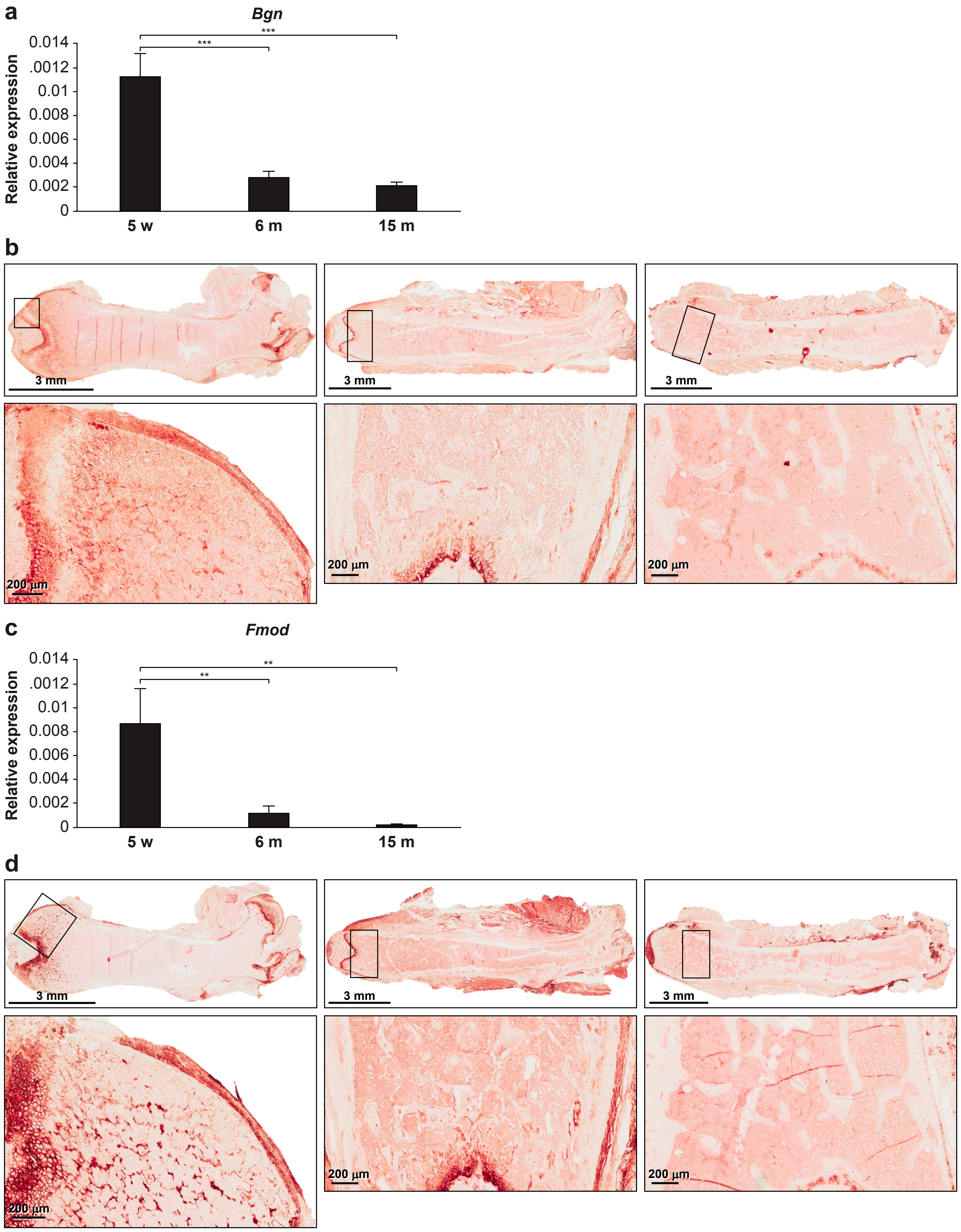

Supplementary Figure 2. Age dependent expression of Bgn and Fmod in bone. (a-b). (A&B). Biglycan expression. (c&d). Fibromodulin expression. (a&c). mRNA expression in BMSCs derived from WT and grown for 14d in osteogenic medium. Data are mean±SE obtained from N=3 cultures per animal from 3-4 different animals per genotype. **, p<0.01; ***, p<0.001 compared with cultures derived from 5w old mice by one-way ANOVA. (b&d) Representative sections of femurs immunohistochemically probed with Bgn (b) and Fmod (d). Upper panels low magnification, bar=3 mm; Boxed areas are shown in higher power at lower panels, bar=200µm. Note in sections from young mice abundant positive staining of both Bgn and Fmod in cell of all developmental stages (e.g. growth plate, the chondro-osteoblast transition of the primary spongiosa, trabeculi and the periosteal lining) and more restricted distribution as well as weaker positive staining as the mice age.

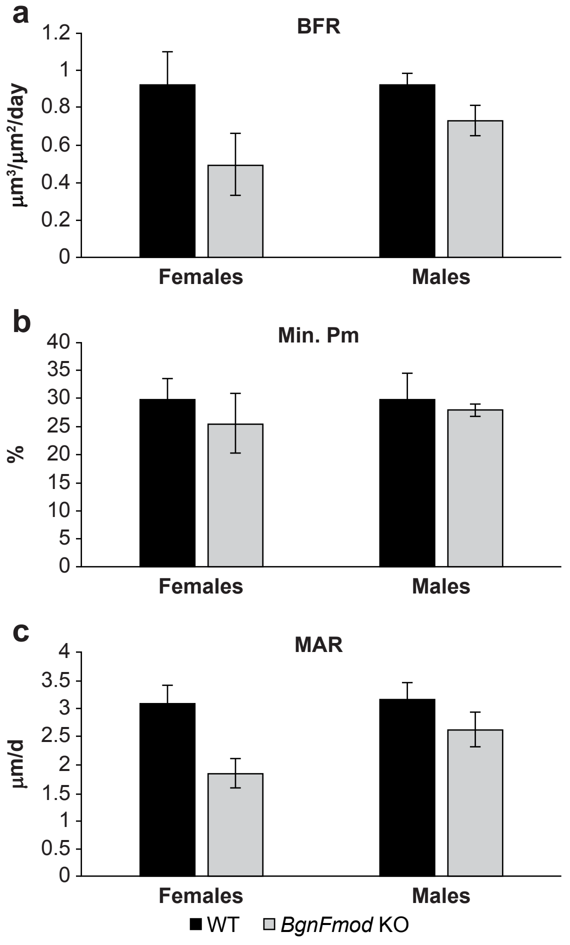

Supplementary Figure 3. Dynamic Histomorphometry at 11w of age. No differences were found in dynamic histomorphometric parameters of 11w old mice (measured in trabecular compartment of distal metaphysis of femur). BFR- bone formation rate; Min.Pm.- mineralizing perimeter; MAR-mineral appositional rate Data are mean±SE obtained from N=4 mice per group.

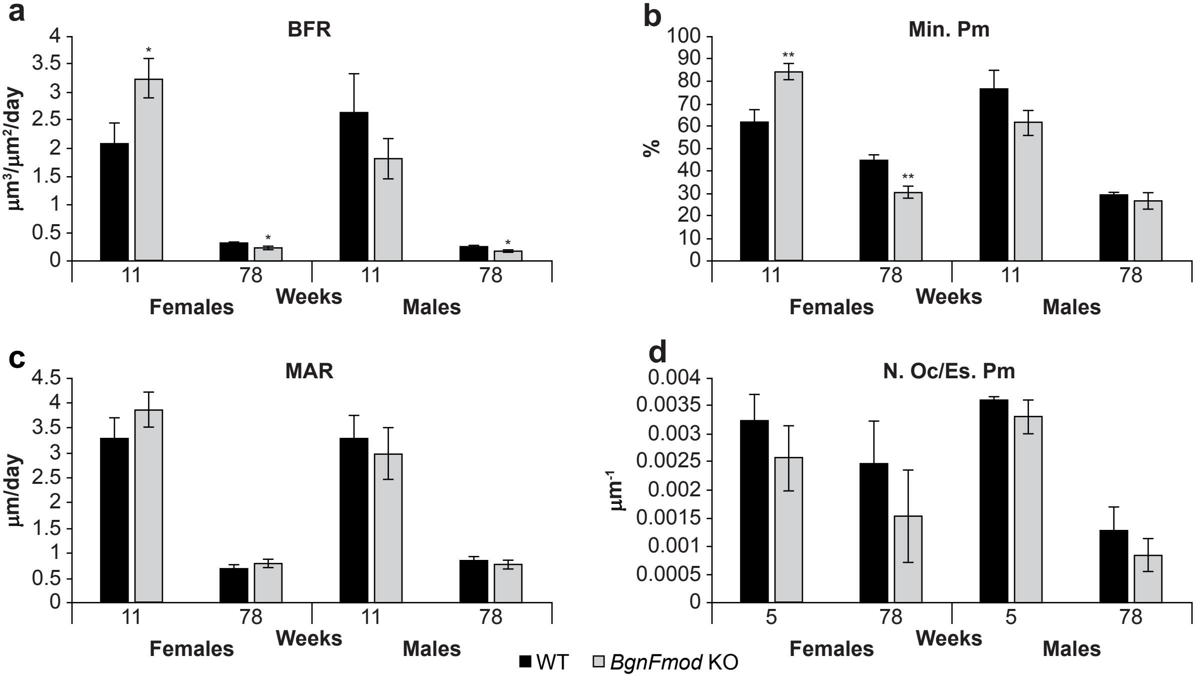

Supplementary Figure 4. Endocortical histomorphometric analysis. (a). Dynamic histomorphometric parameters based on fluorescent visualization of calcein fluorochrome in endocortical perimeter of distal metaphysis of femur. Young female mice presented enhanced bone formation whereas male *BgnFmod* KO mice did not show significant differences compared to age matched WT mice. Both male and female 78w old *BgnFmod* KO mice had markedly decreased endocortical bone formation compared to age matched WT. BFR- bone formation rate; Min.Pm.- mineralizing perimeter; MAR-mineral appositional rate. (b). Quantitative analysis of osteoclast number per endosteal perimeter (N.Oc/Tb.Pm.) measured at distal metaphysis of femur. Data are mean±SE obtained from N=4-6 mice per group. *, $p<0.05$; **, $p<0.01$; ***, $p<0.001$ by unpaired, 2-tailed Student's T test.

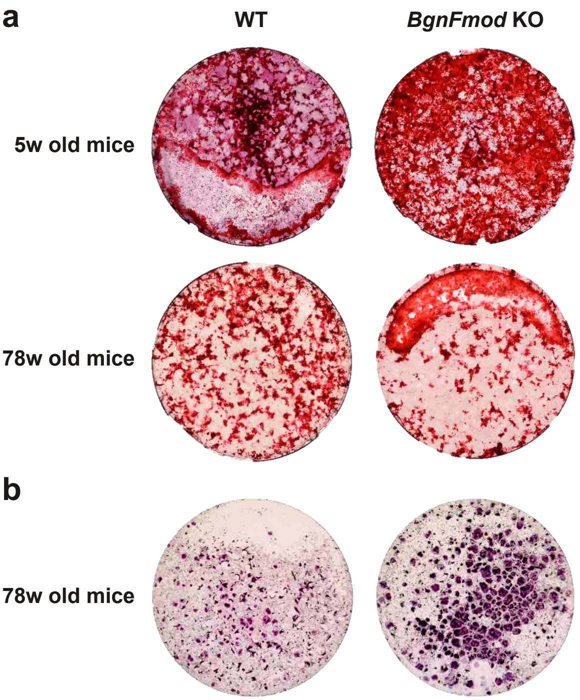

Supplementary Figure 5. Osteoblasts of BgnFmod KO mice lose their heightened bone formation activity with age whereas they retain the enhanced osteoclastogenesis capacity. (a). Representative images of Alizarin red S staining of BMSCs cultures derived from either 5w or 78w old *BgnFmod* KO and WT mice. BMSCs were grown in osteogenic medium for 21d. (b). Representative images of TRAP stained osteoclast cultures derived from 78w old WT or *BgnFmod* KO mice bone marrow.
